# Comparative Efficacy of GLP‐1 Receptor Agonists, Exercise, and Their Combination on Body Composition and Glucolipid Metabolism in Adults With Overweight or Obesity: A Network Meta‐Analysis of Randomized Controlled Trials

**DOI:** 10.1111/obr.70189

**Published:** 2026-07-13

**Authors:** Matteo Vandoni, Alessandro Gatti, Virginia Rossi, Matteo Giuriato, Vittoria Carnevale Pellino, Gianvincenzo Zuccotti, Valeria Calcaterra

**Affiliations:** ^1^ Laboratory of Adapted Motor Activity (LAMA) ‐ Department of Public Health, Experimental Medicine and Forensic Science University of Pavia Pavia Italy; ^2^ National PhD Programme in One Health Approaches to Infectious Diseases and Life Science Research, Department of Public Health, Experimental and Forensic Medicine University of Pavia Pavia Italy; ^3^ Pediatric Department “Vittore Buzzi” Children's Hospital Milan Italy; ^4^ Department of Biomedical and Clinical Science University of Milano Milan Italy; ^5^ Department of Education and Sport Sciences Pegaso University Naples Italy; ^6^ Pediatric and Adolescent Unit, Department of Internal Medicine University of Pavia Pavia Italy

**Keywords:** cardiometabolic, exercise, GLP‐1, obesity, overweight, weight

## Abstract

**Introduction:**

The paper aims to assess the effects of GLP‐1 RAs, structured exercise, and their combination on body composition and cardiometabolic outcomes in adults with overweight or obesity.

**Methods:**

PubMed, Web of Science, Scopus, and EBSCO were searched through April 2026 for English‐language RCTs using terms related to obesity, GLP‐1 RAs, exercise, and glucolipid metabolism outcomes. We included RCTs of adults (18–65 years, BMI ≥ 25 kg/m^2^) without major comorbidities, except for type 2 diabetes, testing GLP‐1 RAs, structured exercise, or both. Of 2508 screened articles, nine studies comprising 1009 participants met eligibility criteria. Participants included 589F/420M, with a weighted mean age of 43.8 ± 12.2 years and weighted mean BMI of 32.6 ± 3.0 kg/m^2^. Risk of bias was assessed using RoB 2. A frequentist random‐effects model calculated SMDs. Primary outcomes were changes in body weight, waist‐to‐hip ratio, and fat mass. Secondary outcomes included fasting glucose, HOMA‐IR, triglycerides, LDL‐C, and HDL‐C. Meta‐regression assessed weight loss as a predictor of metabolic outcomes.

**Results:**

The combination of GLP‐1 receptor agonists and exercise was associated with the greatest effects on weight (SMD −1.04; 95% CI −1.24 to −0.83), fat mass (SMD −1.01; 95% CI −1.27 to −0.75), and waist‐to‐hip ratio (SMD −0.55; 95% CI −0.91 to −0.18) compared with placebo. For fasting glucose, the SMD was −0.49 (95% CI −1.52 to 0.55); for HOMA‐IR, −0.59 (95% CI −0.89 to −0.29). For lipid outcomes, no statistically significant differences were observed for triglycerides or LDL‐C, whereas HDL‐C increased compared with placebo (SMD 0.32; 95% CI 0.04 to 0.61). Confidence in the evidence was high for body weight and fat mass, moderate for waist‐to‐height ratio and most lipid outcomes, and very low for fasting glucose.

**Conclusions:**

Combined GLP‐1 RA and structured exercise interventions improved weight, insulin sensitivity, and selected lipid parameters more than monotherapies or placebo.

## 1 Introduction

The prevalence of obesity has drastically increased over the past decades, exceeding underweight prevalence and reaching pandemic proportions [1]. Specifically in Europe, almost 25% of adults, and up to 33% in some countries, are classified as having obesity [2, 3]. In Italy, the situation is similar; 22% of the women and 20% of the men are affected by obesity, leading to a negative impact on the healthcare system [2, 3]. Obesity is linked to a wide array of health complications, including metabolic, cardiovascular, digestive, respiratory, neurological, musculoskeletal, and infectious disorders [4]. Among these, cardiometabolic conditions are the leading contributors to obesity‐related diseases. Individuals with obesity face nearly a fivefold increase in the risk of developing coronary heart disease, stroke, or diabetes mellitus, with the risk rising to almost fifteenfold for those with class 2 or 3 obesity [5, 6].

Cardiometabolic health is crucial for overall health and longevity, especially for adults with obesity [7, 8]. Indeed, obesity significantly disrupts these processes, leading to conditions such as hypertension, dyslipidaemia, insulin resistance, and type 2 diabetes [9, 10]. Losing just 3 to 5% of body weight can lower risk factors linked to obesity, but individuals with severe obesity or additional health issues are typically advised to aim for a greater reduction, generally over 5 to 15% of their starting weight [11, 12]. Many lifestyle interventions have been developed to counter the obesity pandemic [13, 14], with exercise interventions always being considered a cornerstone for the management of first and second grade obesity in adulthood [15]. Aerobic and resistance training improve not only body weight but also cardiometabolic health [16]: Aerobic training increases energy expenditure and preferentially reduces fat mass, while resistance training preserves or increases lean mass [17], particularly when paired with a controlled diet [18]. Newer evidence further suggests that different modalities of physical exercise exert distinct effects on visceral and intrahepatic fat depots, with high‐intensity interval and combined training showing the most favorable changes in glucose‐intolerant populations [19]. However, lifestyle interventions alone are often not sufficient to achieve the desired weight loss. In recent years, several promising medications have been introduced for weight management. Glucagon‐like peptide‐1 (GLP‐1) receptor agonists have emerged as an innovative pharmacological option, mimicking endogenous GLP‐1 to enhance glucose‐dependent insulin secretion, suppress glucagon release, slow gastric emptying, and reduce appetite via central hypothalamic pathways [20]. In particular, liraglutide and semaglutide result in significant weight loss, often amounting to 10%–15% or more, as well as an improvement in cardiometabolic risk factors [21, 22, 23, 24].

These benefits, however, highlight two limitations that support combining pharmacotherapy with exercise. First, a portion of the weight loss induced by GLP‐1 RAs consists of lean mass. Although the extent varies across studies, lean mass accounted for approximately 10% of total weight loss in STEP‐1 with semaglutide and about 25% in SURMOUNT‐1 with tirzepatide, raising concerns about potential long‐term functional and metabolic consequences [20]. Second, weight loss is often poorly maintained after treatment discontinuation [20]. Resistance training and aerobic exercise directly address both issues, preserving lean body mass and promoting weight maintenance after discontinuation of drug therapy [25], also because physical activity improves endogenous GLP‐1 production [26].

Therefore, recent guidelines emphasize combining pharmacological therapy with structured lifestyle interventions to optimize and sustain treatment outcomes [27]. Indeed, the synergistic potential of exercise when paired with GLP‐1 receptor agonists remains an area of growing interest. However, evidence regarding the effectiveness of pharmacological and structured exercise programs remains contrasting. While individual studies have explored this combination [28], the variability in protocols, populations, and outcome measures has limited the ability to draw clear conclusions regarding its efficacy. To address this gap, we conducted a network meta‐analysis to evaluate the comparative effects of combining exercise interventions with GLP‐1 RAs on cardiometabolic outcomes in adults with obesity. By doing this, our aim is to provide second‐level evidence that may be useful for providing guidelines in the treatment of obesity.

## 2 Materials and Methods

### 2.1 Review Protocol and Registration

This systematic review and network meta‐analysis was conducted and documented in line with the Preferred Reporting Items for Systematic Reviews and Meta‐Analyses (PRISMA 2020 guidelines), the extended PRISMA guidelines for Network Meta‐Analyses, the Cochrane Handbook for Systematic Reviews of Interventions, and the 2021 guidelines from the National Institute for Health and Care Excellence (NICE). The study protocol had been pre‐registered with the PROSPERO international database of systematic reviews (registration ID: CRD420251024708). The detailed checklist is provided in Table S1. The a priori inclusion and exclusion criteria, following the PICOS (Population/Patient, Intervention, Comparison/Control, Outcome and Study design) framework, are detailed in Table S2.

### 2.2 Search Strategy

A comprehensive literature search was conducted in PubMed‐Medline, Web of Science, Scopus and EBSCO up to April 2026. The search targeted studies involving adults and young adults (age range: 18–65 years) examining the effects of GLP‐1 receptor agonists (GLP‐1 RAs), exercise interventions, or their combination on body composition and cardiometabolic outcomes. Keywords included terms related to obesity and overweight (e.g., “BMI,” “body composition,” and “waist circumference”), interventions (e.g., “exercise,” “physical activity,” “GLP‐1 receptor agonist,” and specific GLP‐1 RA drug names), and metabolic or cardiovascular outcomes (e.g., “insulin resistance,” “lipid profile,” “blood pressure,” and “glucose”). The detailed search strings for each database are provided in Table S3. Filters were applied to include only clinical trials, randomized controlled trials, and articles published in English. Two independent reviewers initially screened titles and abstracts for eligibility (A.G. and V.R.), with full texts subsequently retrieved and assessed according to predefined inclusion criteria. Discrepancies were resolved through discussion, with involvement of a third reviewer when necessary (M.V. or V.C.). Additionally, reference lists of relevant articles were manually screened to identify further eligible studies.

### 2.3 Study Selection Criteria

Inclusion criteria were as follows: (i) participants: adults aged 18 to 65 years with overweight or obesity (BMI ≥ 25 kg/m^2^), without known psychiatric, cognitive, or other non‐communicable or acute diseases, except for type 2 diabetes; (ii) interventions: administration of GLP‐1 receptor agonists (GLP‐1 RAs), structured exercise interventions (including aerobic and/or resistance training), or the combination of both; (iii) comparators: placebo, GLP‐1 RAs alone, exercise alone, or no intervention control; (iv) outcomes: changes in body weight, body composition (e.g., fat mass, lean mass, and waist circumference), and cardiometabolic parameters (e.g., fasting glucose, insulin resistance, lipid profile, blood pressure); (v) study design: randomized controlled trials. Trials comparing GLP‐1 RAs with placebo were eligible only when they were conducted within a study framework that also included a structured exercise intervention arm; GLP‐1 RA versus placebo trials without any structured exercise component were excluded because they did not directly address the exercise‐related research question.

### 2.4 Data Extraction and Study Outcomes

A standardized data extraction protocol was used by two independent reviewers (V.R. and A.G.), with discrepancies resolved by a third reviewer (V.C. or M.V.). Extracted data included study characteristics (author, year, country, study design, sample size, age, sex, and baseline BMI), intervention details (GLP‐1 RA type and dose, exercise type, setting, duration, frequency, and intensity), comparator group description, and outcomes of interest. Primary outcomes included changes in body weight and body composition. Secondary outcomes included fasting glucose, insulin resistance (HOMA‐IR), blood pressure, triglycerides, HDL‐C, and LDL‐C. For the network meta‐analysis, mean change‐from‐baseline values and corresponding standard deviations were extracted when available. When change‐from‐baseline data were not reported, post‐intervention values were used. If only confidence intervals were reported, standard deviations were derived. Full details of the data extraction process and outcome prioritization are provided in File S2. A summary of the included studies can be found in Table S4.

### 2.5 Risk of Bias and Quality Assessments

To evaluate the methodological quality of the included studies in the review we used the Revised Cochrane Risk‐of‐Bias Tool for Randomized Trials (ROB2). The ROB2 tool assesses the risk of bias across five key domains: (i) bias arising from the randomization process, (ii) bias due to deviations from intended interventions, (iii) bias due to missing outcome data, (iv) bias in measurement of the outcome, and (v) bias in the selection of the reported result. Each domain was rated as “low risk,” “some concerns,” or “high risk” based on predefined criteria. Assessments were conducted independently by two reviewers (A.G. and V.R.), with differences resolved through discussion or consultation with a third reviewer (M.V. or V.C.) if necessary.

### 2.6 Confidence in Evidence Assessment

The confidence in the evidence for the network estimates was assessed using the Confidence in Network Meta‐Analysis framework (CINeMA), which is based on the GRADE approach for network meta‐analysis. Confidence was evaluated across six domains: within‐study bias, reporting bias, indirectness, imprecision, heterogeneity, and incoherence. Within‐study bias was informed by the RoB 2 assessment. Imprecision was judged according to the width of the 95% confidence intervals and whether they crossed or approached the line of no effect. Heterogeneity was assessed using *τ* ^2^, Cochran's *Q*, and *I* ^2^ statistics, while incoherence was evaluated by examining disagreement between direct and indirect evidence where available. The overall confidence rating for each comparison was classified as high, moderate, low, or very low.

### 2.7 Statistical Analysis

Data from randomized controlled trials were analyzed using a standardized mean difference (SMD) approach to assess intervention effects across the network of comparisons. Post‐pre intervention changes were calculated for each intervention group where available; if change‐from‐baseline values were not reported, post‐intervention means were used. A pooled baseline standard deviation was used to standardize effect sizes across studies. SMDs were computed, implementing Hedges' *g*. A frequentist network meta‐analysis (NMA) was conducted using a random‐effects model to account for between‐study heterogeneity, applying inverse variance weighting for each pairwise comparison. Network geometry was visualized using network plots, with node sizes proportional to the number of participants and edge thickness proportional to the number of studies informing each comparison. Forest plots were generated to present pooled SMDs with 95% confidence intervals (CIs), along with *P*‐scores to rank interventions according to their relative efficacy. Prediction intervals were calculated to illustrate the range of expected effects in future studies. Heterogeneity was assessed using Cochran's *Q* test and the *I* ^2^ statistic, with *I* ^2^ categorized as: might not be important (0%–40%), moderate (30%–60%), substantial (50%–90%), and considerable (75%–100%). League tables presenting all pairwise comparisons with 95% CIs were produced. Transitivity was assessed by evaluating two main criteria (as previously done by other network meta‐analyses [20]): (i) the extent to which participants in the included studies could have plausibly been randomized to any of the interventions in the network, and (ii) the distribution of potential effect modifiers across treatment comparisons. To address the first, we examined trial eligibility criteria and consulted with clinical experts to confirm the appropriateness of the comparisons. For the second, we reviewed the included studies for balance in pre‐specified variables, such as mean age, sex ratio, intervention duration, follow‐up period, and risk of bias. The distribution of these factors was deemed sufficiently similar across comparisons to support the validity of the transitivity assumption. Comparison‐adjusted funnel plots were also generated to assess the potential for small‐study effects; these are presented in the Supporting Information (from Figures S1–S8). Treatment rankings were calculated using SUCRA values derived from the cumulative ranking probabilities for each intervention and outcome. SUCRA values were expressed as percentages, ranging from 0% to 100%, with higher values indicating a greater probability of being among the most effective interventions, and were reported in Table S5 together with the corresponding intervention rank. Exploratory univariable moderation analyses were also conducted to assess whether diabetes status, intervention duration, and percentage of women moderated treatment effects. These analyses were performed separately for each outcome using SMD estimates, with GLP‐1 RAs plus exercise as the reference intervention. Moderator effects were reported as SMD *β* coefficients with 95% CIs and *p* values; duration and percentage of women were scaled per 10‐unit increase (Table S6).

We also conducted meta‐regression analyses to investigate the relationship between changes in body weight and other clinical outcomes (from Figures S9–S13). All analyses were conducted using R software (Version 4.4), with netmeta used for NMA and visualization, and metafor package for the meta‐regression.

## 3 Results

### 3.1 Synthesis of Studies Found in the Systematic Search

**FIGURE 1:**
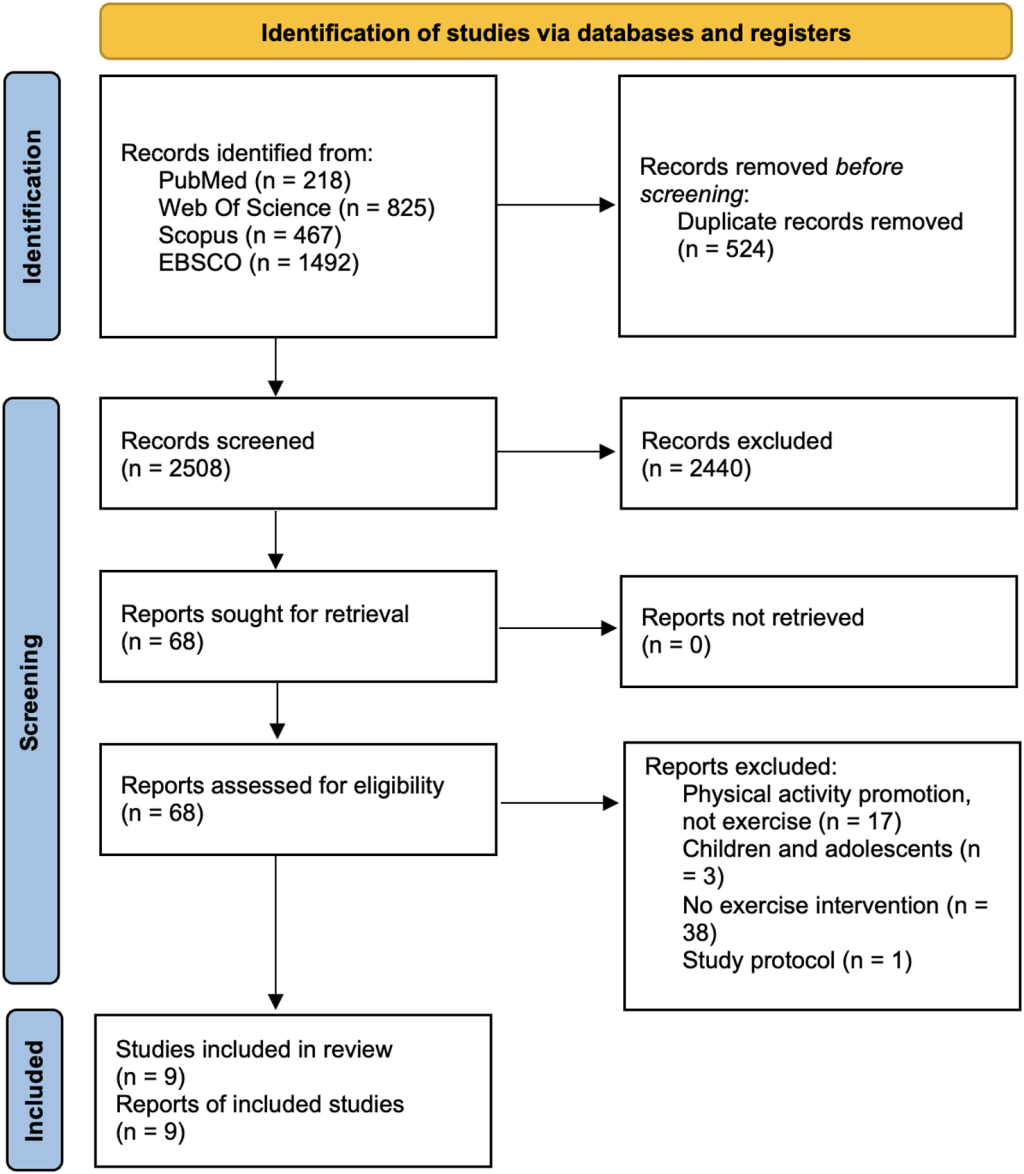
Preferred Reporting Items for Systematic Reviews and Meta‐Analyses (PRISMA) flow diagram.

The systematic search and study selection process is illustrated in the PRISMA flow diagram (Figure 1). Following the removal of duplicate and not in English records, a total of 2508 studies were screened by title and abstract. Of these, 68 full‐text articles were further assessed for eligibility. The primary reasons for excluding studies at this stage were: (i) the intervention was based on a physical activity promotion and not an exercise intervention (17 studies); (ii) studies focused on children and adolescents (thee studies); (iii) intervention focused only on GLP‐1 RAs and on placebo (38 studies); and (iv) study protocol (*n* = 1). Therefore, nine studies that met the inclusion criteria were selected for this systematic review, and all nine studies were included in the meta‐analyses. The included studies involved adult participants who were generally middle‐aged and had BMI values within the obesity range. Reported sample sizes ranged from 31 to 195 participants per study. Across the studies we included 1009 participants, including 420 men and 589 women. Mean age ranged from 42 to 59 years, while mean BMI ranged from 30.8 to 34.8 kg/m^2^. Among the included studies, only two also considered patients with type 2 diabetes.

Risk‐of‐bias assessment for every study is shown in Figure S14, while the overall risk‐of‐bias is displayed in Figure S15. Overall, most of the studies were classified as at “low” risk of bias, with two studies considered as “some concerns.” Most of the concerns were related to problems in the statistical analyses performed.

### 3.2 Structure of the Network

**FIGURE 2:**
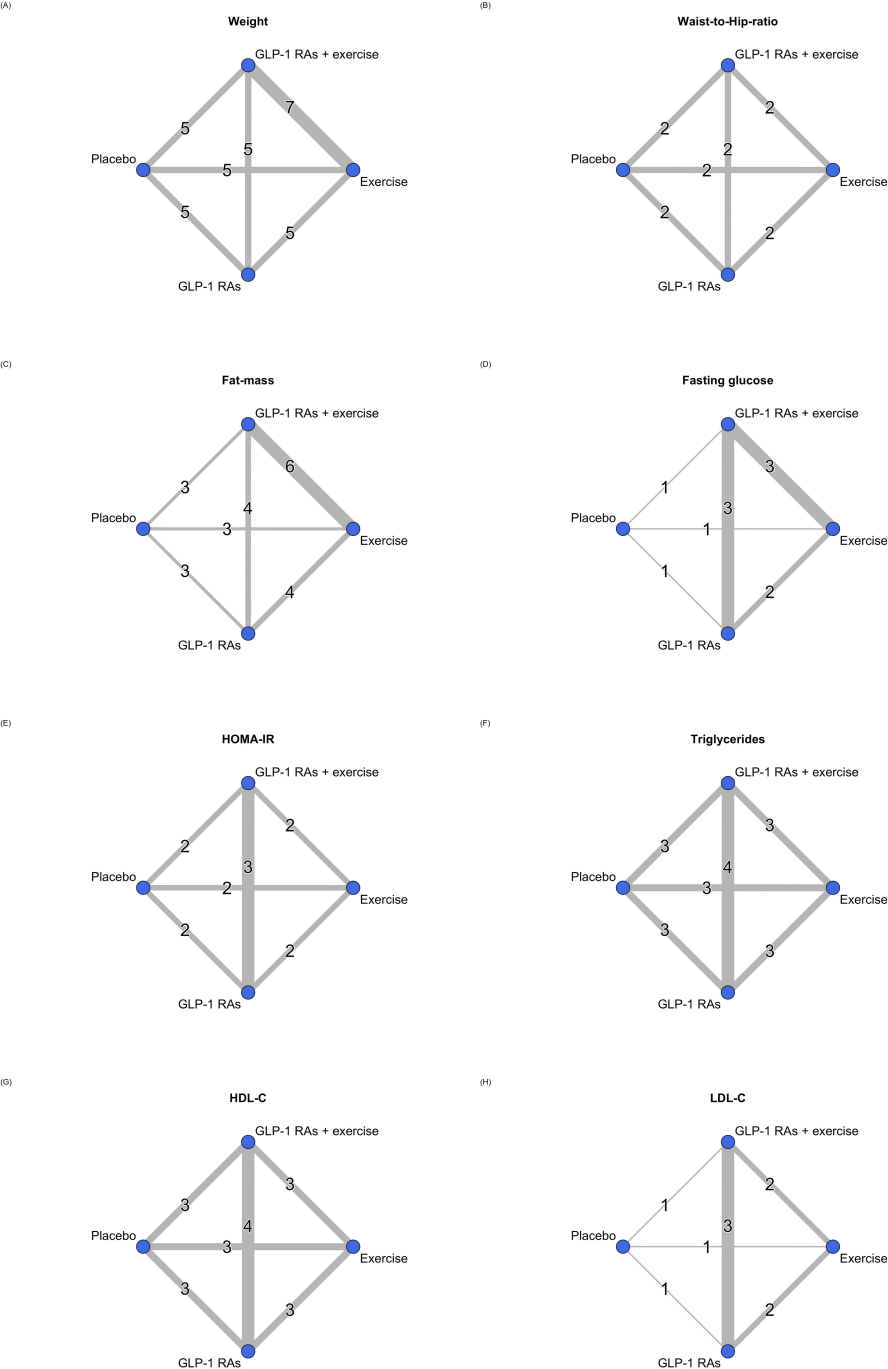
Network diagrams illustrating the available comparisons for anthropometric and metabolic outcomes. Line thickness reflects the number of studies directly comparing each intervention pair, while node size corresponds to the total number of participants in each treatment group. Each node indicates a treatment group: placebo, exercise, GLP‐1 receptor agonists (GLP‐1 RAs), and their combination. The outcomes evaluated include (A) weight, (B) waist‐to‐hip ratio, (C) fat mass, (D) fasting glucose, (E) homeostatic model assessment of insulin resistance (HOMA‐IR), (F) triglycerides, (G) high‐density lipoprotein cholesterol (HDL‐C), and (H) low‐density lipoprotein cholesterol (LDL‐C).

Figure 2 illustrates the network graph for the primary outcomes across the included interventions: placebo, GLP‐1 RAs, exercise, and their combination. Each node represents an intervention, and the size of the node reflects the number of participants involved. The connecting lines indicate direct comparisons, with line thickness proportional to the number of studies informing each comparison. The networks for outcomes such as weight, waist‐to‐hip ratio, fat mass, and HOMA‐IR are fully connected, allowing for both direct and indirect comparisons across interventions. In contrast, lipid outcomes (i.e., HDL‐C and LDL‐C) and fasting glucose display reduced connectivity, primarily relying on direct comparisons between select pairs, which may limit the robustness of indirect estimates for those outcomes.

### 3.3 Different Types of Intervention Effects on Anthropometric Characteristics

**FIGURE 3:**
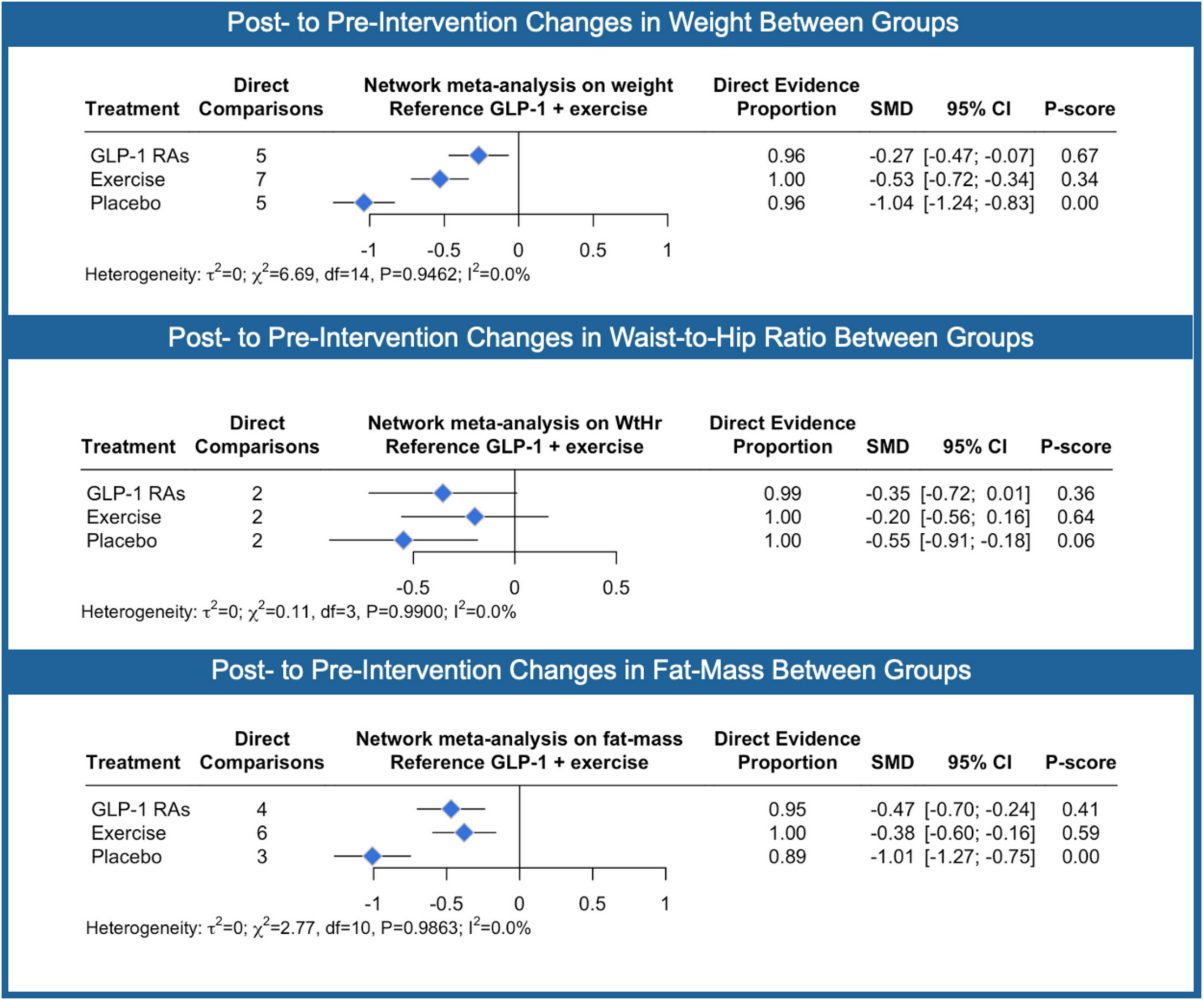
Network meta‐analysis results comparing the effects of GLP‐1 receptor agonists (GLP‐1 RAs), exercise, and placebo against the reference group (GLP‐1 RAs + exercise) on (top) body weight, (middle) waist‐to‐hip ratio, and (bottom) fat mass. The number of direct comparisons for each treatment group is listed, alongside the standardized mean difference (SMD), 95% confidence intervals (CI), and *P*‐scores, which range from 0 (*least effective*) to 1 (*most effective*). Proportion values indicate the contribution of direct evidence to the network estimates. Heterogeneity statistics (*τ* ^2^, *χ* ^2^, *I* ^2^) are reported below each graph to assess consistency across included studies.

Based on the network meta‐analysis, the combination of GLP‐1 RAs and exercise was associated with the greatest and statistically significant reduction in weight compared with placebo (SMD −1.04, 95% CI −1.24 to −0.83; Figure 3). Moreover, the combination group obtained greater significant reductions in weight compared with both GLP‐1 RAs alone (SMD −0.27, 95% CI −0.47 to −0.07) and exercise alone (SMD −0.53, 95% CI −0.72 to −0.34). For waist‐to‐hip ratio, the combination intervention showed a significant advantage over placebo (SMD −0.55, 95% CI −0.91 to −0.18), whereas no statistically significant difference was observed compared with GLP‐1 RAs alone (SMD −0.35, 95% CI −0.72 to 0.01). We found no statistically significant differences between GLP‐1 RAs + exercise and exercise alone (SMD −0.20, 95% CI −0.56 to 0.16). A similar pattern was observed for fat mass, with the largest difference seen in the combination group versus placebo (SMD −1.01, 95% CI −1.27 to −0.75). Moreover, the combination group compared to the GLP‐1 RAs alone obtained a greater significant reduction (SMD −0.47, 95% CI −0.70 to −0.24) and showed a greater significant reduction compared with exercise alone (SMD −0.38, 95% CI −0.60 to −0.16). Heterogeneity was low across outcomes, with *I* ^2^ = 0.0% for weight, waist‐to‐hip ratio, and fat mass. Treatment rankings based on SUCRA values showed that GLP‐1 RAs + exercise ranked first for all anthropometric outcomes, including weight, WHtR, and fat mass (Table S5). Exploratory univariable moderation analyses suggested that diabetes status, intervention duration, and percentage of women moderated the effect on weight, while diabetes status and intervention duration moderated the effect on fat mass (Table S6).

The league table for the comparisons between groups is presented in Table S7.

### 3.4 Different Types of Intervention Effects on Glycemic Profile

**FIGURE 4:**
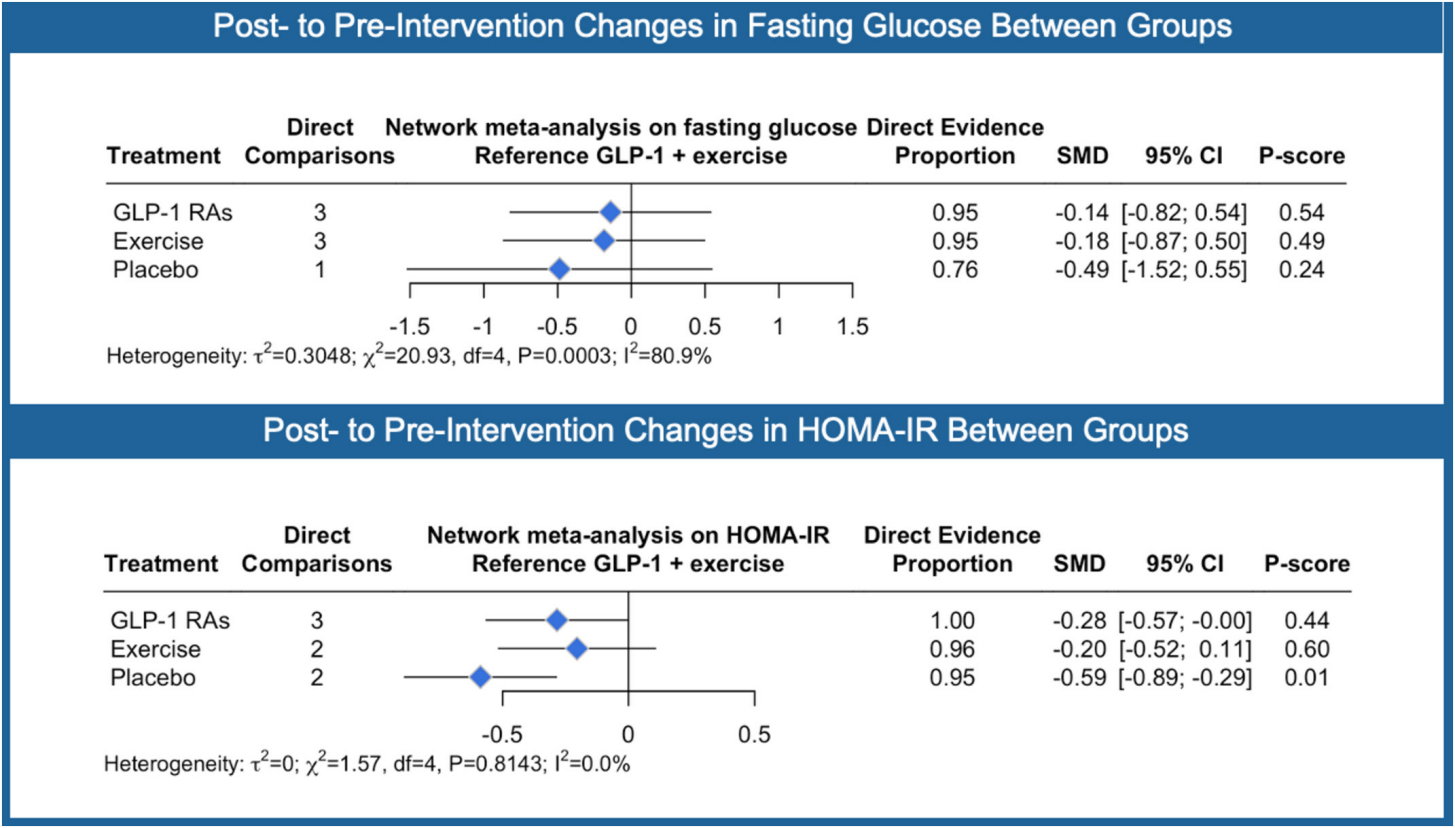
Network meta‐analysis results comparing the effects of GLP‐1 receptor agonists (GLP‐1 RAs), exercise, and placebo against the reference group (GLP‐1 RAs + exercise) on (top) fasting glucose, and (bottom) homeostatic model assessment of insulin resistance (HOMA‐IR). The number of direct comparisons for each treatment group is listed, alongside the standardized mean difference (SMD), 95% confidence intervals (CI), and P‐scores, which range from 0 (*least effective*) to 1 (*most effective*). Proportion values indicate the contribution of direct evidence to the network estimates. Heterogeneity statistics (*τ* ^2^, *χ* ^2^, *I* ^2^) are reported below each graph to assess consistency across included studies.

Based on the network meta‐analysis, the combination of GLP‐1 RAs and exercise showed no statistically significant difference in fasting glucose compared with placebo (SMD −0.49, 95% CI −1.52 to 0.55; Figure 4). However, no statistically significant differences were observed between the combination group and either GLP‐1 RAs alone (SMD −0.14, 95% CI −0.82 to 0.54) or exercise alone (SMD −0.18, 95% CI −0.87 to 0.50). For HOMA‐IR, the combination group also showed the largest and significant improvement compared with placebo (SMD −0.59, 95% CI −0.89 to −0.29). A statistically significant difference was also observed when comparing the combination to GLP‐1 RAs alone (SMD −0.28, 95% CI −0.57 to −0.00), while the comparison with exercise alone did not reach significance (SMD −0.20, 95% CI −0.52 to 0.11). Heterogeneity was high for fasting glucose (*I* ^2^ = 80.9%), indicating substantial variability across studies, whereas no heterogeneity was detected for HOMA‐IR (*I* ^2^ = 0.0%). Treatment rankings based on SUCRA values showed that GLP‐1 RAs + exercise ranked first for both fasting glucose and HOMA‐IR, with SUCRA values of 72.6% and 96.1%, respectively (Table S5). Exploratory univariable moderation analyses did not identify significant moderators for fasting glucose or HOMA‐IR, including diabetes status, intervention duration, and percentage of women (Table S6).

The league table for the comparisons between groups is presented in the Table S8.

### 3.5 Different Types of Interventions Effects on Lipid Profile

**FIGURE 5:**
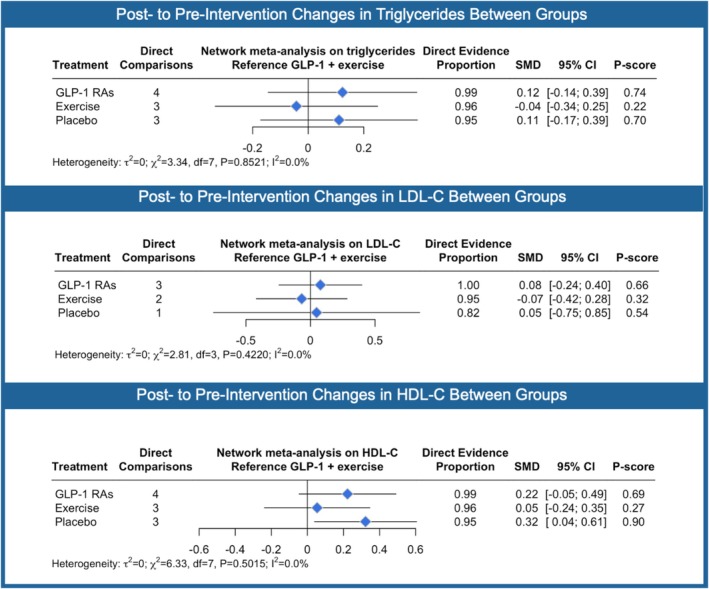
Network meta‐analysis results comparing the effects of GLP‐1 receptor agonists (GLP‐1 RAs), exercise, and placebo against the reference group (GLP‐1 RAs + exercise) on (top) triglycerides glucose, (middle) low‐density lipoprotein cholesterol (LDL‐C) and (bottom) high‐density lipoprotein cholesterol (HDL). The number of direct comparisons for each treatment group is listed, alongside the standardized mean difference (SMD), 95% confidence intervals (CI), and P‐scores, which range from 0 (least effective) to 1 (most effective). Proportion values indicate the contribution of direct evidence to the network estimates. Heterogeneity statistics (*τ* ^2^, *χ* ^2^, *I* ^2^) are reported below each graph to assess consistency across included studies.

Based on the network meta‐analysis, no statistically significant differences were found in triglyceride levels between GLP‐1 RAs + exercise and any of the individual interventions, including GLP‐1 RAs alone (SMD 0.12, 95% CI −0.14 to 0.39), exercise alone (SMD −0.04, 95% CI −0.34 to 0.25), or placebo (SMD 0.11, 95% CI −0.17 to 0.39; Figure 5). For LDL‐C, no statistically significant differences were observed between the combination group and placebo (SMD −0.05, 95% CI −0.75 to 0.85), or GLP‐1 RAs alone (SMD 0.08, 95% CI −0.24 to 0.40) or exercise alone (SMD −0.07, 95% CI −0.42 to 0.28). In contrast, for HDL‐C the combination group obtained a greater increase compared to placebo (SMD 0.32, 95% CI 0.04 to 0.61). However, the combination has not improved HDL‐C statistically more than the exercise group (SMD 0.05, 95% CI −0.44 to 0.54) or GLP‐1 RAs alone (SMD 0.22, 95% CI −0.05 to 0.49). The estimated *I* ^2^ was 0.0% for triglycerides, LDL‐C, and HDL‐C, suggesting negligible observed between‐study heterogeneity across lipid outcomes. Treatment rankings based on SUCRA values showed that GLP‐1 RAs ranked first for triglycerides and LDL‐C, whereas GLP‐1 RAs + exercise ranked first for HDL‐C (Table S5). Exploratory univariable moderation analyses did not identify significant moderators for triglycerides, LDL‐C, or HDL‐C (Table S6). The league table for the comparisons between groups is presented in Table S9.

### 3.6 Confidence in the Evidence

Table 1 presents the confidence in the evidence assessed using the CINeMA framework. For anthropometric outcomes, the confidence rating was high for body weight and fat mass, whereas waist‐to‐hip ratio showed moderate confidence because some comparisons had confidence intervals crossing or approaching the line of no effect. Among glycemic outcomes, HOMA‐IR showed moderate to high confidence, whereas fasting glucose was rated as very low because of major concerns regarding imprecision and heterogeneity. For lipid outcomes, confidence ranged from moderate to low. Triglycerides and HDL‐C were generally rated as moderate, mainly because confidence intervals crossed or were close to the null value. LDL‐C showed moderate confidence for comparisons with GLP‐1 RAs and exercise, but low confidence for the comparison with placebo because the estimate was highly imprecise. Across outcomes, within‐study bias was judged as having no concerns because the RoB2 assessment indicated low risk of bias across the included randomized controlled trials.

**TABLE 1:** Summary of mixed evidence confidence ratings (CRs) using the CINeMA framework.

Outcome	Comparison	*k*	WsB	Reporting bias	Indirectness	Imprecision	Heterogeneity	Incoherence	CR
Weight	GLP‐1 RAs + exercise: GLP‐1 RAs	5	No concerns	Low risk	No concerns	No concerns	No concerns	No concerns	High
	GLP‐1 RAs + exercise: Exercise	7	No concerns	Low risk	No concerns	No concerns	No concerns	No concerns	High
	GLP‐1 RAs + exercise: Placebo	5	No concerns	Low risk	No concerns	No concerns	No concerns	No concerns	High
Waist‐to‐hip ratio	GLP‐1 RAs + exercise: GLP‐1 RAs	2	No concerns	Low risk	No concerns	Some concerns	No concerns	No concerns	Moderate
	GLP‐1 RAs + exercise: Exercise	2	No concerns	Low risk	No concerns	Some concerns	No concerns	No concerns	Moderate
	GLP‐1 RAs + exercise: Placebo	2	No concerns	Low risk	No concerns	Some concerns	No concerns	No concerns	Moderate
Fat mass	GLP‐1 RAs + exercise: GLP‐1 RAs	4	No concerns	Low risk	No concerns	No concerns	No concerns	No concerns	High
	GLP‐1 RAs + exercise: Exercise	6	No concerns	Low risk	No concerns	No concerns	No concerns	No concerns	High
	GLP‐1 RAs + exercise: Placebo	3	No concerns	Low risk	No concerns	No concerns	No concerns	No concerns	High
Fasting glucose	GLP‐1 RAs + exercise: GLP‐1 RAs	3	No concerns	Low risk	No concerns	Major concerns	Major concerns	No concerns	Very low
	GLP‐1 RAs + exercise: Exercise	3	No concerns	Low risk	No concerns	Major concerns	Major concerns	No concerns	Very low
	GLP‐1 RAs + exercise: Placebo	1	No concerns	Low risk	No concerns	Major concerns	Major concerns	No concerns	Very low
HOMA‐IR	GLP‐1 RAs + exercise: GLP‐1 RAs	3	No concerns	Low risk	No concerns	Some concerns	No concerns	No concerns	Moderate
	GLP‐1 RAs + exercise: Exercise	2	No concerns	Low risk	No concerns	Some concerns	No concerns	No concerns	Moderate
	GLP‐1 RAs + exercise: Placebo	2	No concerns	Low risk	No concerns	No concerns	No concerns	No concerns	High
Triglycerides	GLP‐1 RAs + exercise: GLP‐1 RAs	4	No concerns	Low risk	No concerns	Some concerns	No concerns	No concerns	Moderate
	GLP‐1 RAs + exercise: Exercise	3	No concerns	Low risk	No concerns	Some concerns	No concerns	No concerns	Moderate
	GLP‐1 RAs + exercise: Placebo	3	No concerns	Low risk	No concerns	Some concerns	No concerns	No concerns	Moderate
LDL‐C	GLP‐1 RAs + exercise: GLP‐1 RAs	3	No concerns	Low risk	No concerns	Some concerns	No concerns	No concerns	Moderate
	GLP‐1 RAs + exercise: Exercise	2	No concerns	Low risk	No concerns	Some concerns	No concerns	No concerns	Moderate
	GLP‐1 RAs + exercise: Placebo	1	No concerns	Low risk	No concerns	Major concerns	No concerns	No concerns	Low
HDL‐C	GLP‐1 RAs + exercise: GLP‐1 RAs	4	No concerns	Low risk	No concerns	Some concerns	No concerns	No concerns	Moderate
	GLP‐1 RAs + exercise: Exercise	3	No concerns	Low risk	No concerns	Some concerns	No concerns	No concerns	Moderate
	GLP‐1 RAs + exercise: Placebo	3	No concerns	Low risk	No concerns	Some concerns	No concerns	No concerns	Moderate

*Note:* Confidence ratings were assessed using the CINeMA framework, which is based on the GRADE approach for network meta‐analysis. Within‐study bias was judged according to the RoB2 assessment. Imprecision was judged according to the width of the 95% confidence intervals and whether they crossed or approached the line of no effect. Heterogeneity was judged using tau‐squared, chi‐squared, and *I*‐squared statistics. Incoherence was considered as having no concerns when no relevant disagreement between direct and indirect evidence was detected; this judgment should be checked against the final inconsistency/coherence diagnostics.

Abbreviations: CR, confidence rating; GLP‐1 RAs, glucagon‐like peptide‐1 receptor agonists; HDL‐C, high‐density lipoprotein cholesterol; HOMA‐IR, homeostatic model assessment of insulin resistance; *k*, number of direct comparisons/studies providing direct evidence; LDL‐C, low‐density lipoprotein cholesterol; WsB, within‐study bias.

## 4 Discussion

This systematic review and network meta‐analysis examined the effects of GLP‐1 RAs, structured exercise interventions, and their combination on body composition and cardiometabolic outcomes in adults with obesity. Overall, our findings suggest the superiority of a combined approach, which was associated with greater effects than monotherapies or a placebo intervention on body weight and metabolic parameters. The observed reductions also in waist‐to‐hip ratio and fat mass compared to placebo may indicate that beyond mere caloric deficit, the combined intervention could act through depot‐specific fat mobilization, potentially affecting visceral adiposity to a greater extent [29, 30].

As for glycemic outcomes, combination therapy was associated with higher benefits for insulin resistance, as evidenced by a significant improvement in HOMA‐IR compared to placebo and GLP‐1 RAs alone. Although fasting glucose levels decreased similarly in the combination and GLP‐1 RA groups, the additional improvement in HOMA‐IR suggests that exercise may uniquely contribute to enhancing insulin sensitivity. Unlike GLP‐1 RAs, which primarily improve glycemic control via enhanced insulin secretion and appetite suppression [31], exercise exerts peripheral effects on glucose uptake and mitochondrial function [32]. This is consistent with exercise‐only meta‐analyses in adults with concurrent type 2 diabetes and obesity, in which aerobic training and the combined aerobic‐plus‐resistance training improved glycemic control relative to standard care [33, 34], and in which higher‐intensity protocols reduced fasting insulin and HOMA‐IR specifically [35, 36]. Together, these distinct mechanisms appear to complement one another, supporting the use of combined therapy for more comprehensive metabolic benefits.

Lipid‐related outcomes were more variable and less stable than glycemic effects. LDL‐C decreased significantly with combination therapy compared to placebo but not compared to monotherapies. HDL‐C improved mainly in groups receiving exercise, which may highlight the key role of exercise in HDL regulation [37, 38]. A similar pattern has also been observed in studies focusing exclusively on physical exercise, in which combined aerobic and resistance training had a positive effect on lipid metabolism [39] and high‐intensity training selectively increased HDL cholesterol in participants with comorbidities [36], which is consistent with the interpretation that the exercise component may underlie the lipid benefits observed in the combined treatment group. No significant changes in triglycerides were observed. These inconsistent effects may reflect the limited direct comparisons, which likely reduced the precision of network estimates. This emphasizes the need for further targeted trials to determine the effects of interventions on the lipid profile.

To explore this further, we conducted meta‐regression analyses using total body weight change as a predictor. These analyses demonstrated significant associations between greater weight loss and improvements in waist‐to‐hip ratio, fat mass, HOMA‐IR, and both LDL‐C and HDL‐C. These findings suggest that the extent of weight loss achieved, especially in the combination group, may play a central role in mediating broader metabolic benefits. These associations are consistent with weight loss being an important contributor to cardiometabolic improvements, particularly in outcomes related to adiposity, insulin resistance, and lipid balance, although such associations at the study level cannot establish causality at the individual level. However, the consistent superiority of the combined intervention also points to possible synergistic effects that may go beyond weight reduction alone. In particular, some meta‐analyses focusing exclusively on physical exercise have documented improvements in insulin resistance and various cardiometabolic indices even in the absence of significant weight loss [35, 36], which is consistent with the view that physical exercise provides benefits that are, at least in part, independent of body weight.

Indeed, these additive benefits likely stem from the distinct yet complementary physiological effects of GLP‐1 RA and exercise. GLP‐1 RAs promote weight loss primarily through appetite suppression, delayed gastric emptying, and enhanced insulin secretion [40]. On a molecular level, GLP‐1 RAs activate signaling pathways such as cAMP/PKA, PI3K/Akt, and MAPK, which are involved in enhancing pancreatic beta‐cell responsiveness, reducing hepatic glucose production, and improving endothelial function [41, 42, 43]. In contrast, structured exercise improves insulin sensitivity, glucose uptake, and lipid metabolism via upregulation of GLUT‐4 transporters, activation of AMP‐activated protein kinase (AMPK), and increased mitochondrial biogenesis in skeletal muscle [44, 45]. Resistance training, specifically, contributes to the preservation of lean body mass by stimulating muscle protein synthesis and suppressing proteolytic pathways [46]. The combination of these mechanisms may help account for the enhanced glycemic control and body composition improvements seen in combined interventions. Notably, GLP‐1 RAs may also influence muscle tissue indirectly by reducing ectopic fat deposition and systemic inflammation [47], while exercise can potentiate the glucose‐lowering effects of GLP‐1 RAs by improving peripheral insulin action [30].

Several of the included trials support this mechanistic synergy. For instance, Jensen et al. [48] showed that combining aerobic exercise with liraglutide led to a 122% improvement in beta‐cell function, alongside significant reductions in postprandial glucose and glucagon levels, which are benefits greater than those observed with either intervention alone. Only the combination significantly improved the disposition index, a key marker of beta‐cell function adjusted for insulin sensitivity [49]. Similarly, Ingersen et al. [50] found an increase in insulin secretion in the combination group approximately sevenfold higher than with training alone and was not attributable to weight loss. These findings are consistent with GLP‐1 RAs and exercise acting through distinct but complementary mechanisms: GLP‐1 RAs primarily enhancing insulin secretion, and exercise improving insulin sensitivity and beta‐cell responsiveness, which supports concurrent use in the management of cardiometabolic health in obesity. Beyond metabolic endpoints, a recent secondary analysis of one of the included trials demonstrated that the combination of exercise and liraglutide produced clinically meaningful gains in physical functional performance and cardiorespiratory fitness during weight maintenance, whereas GLP‐1 RA treatment alone did not improve these fitness outcomes [51]. This observation is clinically relevant, as it suggests that the functional and cardiorespiratory benefits of the combined approach may be attributable to the exercise component and may not be achieved by pharmacotherapy alone.

An additional and important consideration is the known loss of lean body mass (LBM) associated with most weight‐loss interventions. Reductions in LBM are estimated at 15%–25% with lifestyle interventions, 25%–40% with GLP‐1 RAs, and approximately 31% following bariatric surgery [52, 53]. Given the central role of skeletal muscle in metabolic regulation, mobility, and long‐term weight maintenance, this represents a relevant clinical concern. Physical activity, particularly resistance training, may play a key role in mitigating LBM loss. Evidence indicates that resistance training during caloric restriction can preserve 50%–95% of lean mass and support bone density [54]. The relevance of preserving muscle function is further underscored by evidence that combined exercise improves not only body composition but also muscle strength and physical function alongside pharmacological weight loss [39, 51]. However, due to the limited data available from the studies included in this network meta‐analysis, it was not possible to analyze changes in lean mass across the interventions. Consequently, the need for future studies is evident, as they will provide the necessary evidence to determine whether combining exercise and pharmacological interventions results in the maintenance or improvement of lean mass.

Although this study was conducted with methodological rigor, several limitations should be considered. First, the number of eligible studies was limited, specifically for lipid profiles, reducing the confidence of indirect comparisons. Second, the considerable heterogeneity observed in both glycemic and lipid outcomes may reflect a complex interplay of factors, including variations in intervention duration, exercise intensity, and population characteristics. This variability is further compounded by biological influences (e.g., differential effects on metabolic pathways and lipid fractions) and behavioral differences (e.g., adherence and individual response to exercise). We did not include GLP‐1 RA versus placebo trials that lacked a structured exercise arm. Although their inclusion may have improved the precision of pharmacological comparisons, it would have broadened the scope of the network and may have increased clinical and methodological heterogeneity. Lastly, due to insufficient data availability we could not analyze the changes in lean mass, which should be considered a crucial outcome to evaluate the feasibility of these interventions.

This study has several notable strengths. It is the first network meta‐analysis to comprehensively evaluate the combined effects of GLP‐1 receptor agonists and structured exercise on both body composition and a wide range of cardiometabolic outcomes in adults with obesity. The use of a network meta‐analytic approach allowed for the integration of both direct and indirect evidence, increasing the precision of comparative estimates across multiple interventions. Additionally, the inclusion of meta‐regression analyses enabled the identification of weight loss as a key mediator of metabolic improvements, providing relevant mechanistic insight. The study adhered to rigorous methodological standards, including a robust risk of bias assessment, publication bias evaluation, and a comprehensive search across five major databases (PubMed‐Medline, Web of Science, Scopus, and EBSCO).

In conclusion, our network meta‐analysis suggests the growing consensus that combining GLP‐1 RAs with structured exercise may offer the most comprehensive benefits in adults with obesity. While GLP‐1 RAs appear to provide strong glycemic control and exercise seems essential for body composition and functional health, their combination was associated with superior and more balanced outcomes across the interventions compared in this network. These findings point to the importance of a multidimensional, personalized approach to obesity management that prioritizes not only weight loss but overall cardiovascular and metabolic health.

## Funding

The authors have nothing to report.

## Conflicts of Interest

The authors declare no conflicts of interest.

## Supporting information




**Figure S1:** Funnel plot of network meta‐analysis depicting the relationship between e < ect size versus standard error for the e < ect of di<erent therapies on weight.
**Figure S2:**. Funnel plot of network meta‐analysis depicting the relationship between e < ect size versus standard error for the e < ect of di<erent therapies on Waist‐to‐hip‐ratio (WtHr).
**Figure S3:** Funnel plot of network meta‐analysis depicting the relationship between e < ect size versus standard error for the e < ect of di<erent therapies on fat‐mass.
**Figure S4:** Funnel plot of network meta‐analysis depicting the relationship between e < ect size versus standard error for the e < ect of di<erent therapies on fasting glucose.
**Figure S5:** Funnel plot of network meta‐analysis depicting the relationship between e < ect size versus standard error for the e < ect of di<erent therapies on homeostatic model assessment of insulin resistance (HOMA‐IR).
**Figure S6:** Funnel plot of network meta‐analysis depicting the relationship between e < ect size versus standard error for the e < ect of di<erent therapies on triglycerides.
**Figure S7:** Funnel plot of network meta‐analysis depicting the relationship between e < ect size versus standard error for the e < ect of di<erent therapies on LDL‐C.
**Figure S8:** Funnel plot of network meta‐analysis depicting the relationship between e < ect size versus standard error for the e < ect of di<erent therapies on HDL‐C.
**Figure S9:** Meta‐regression analysis demonstrating the association of total body weight reduction with changes in waist‐to‐hip‐ratio (WtHr).
**Figure S10:** Meta‐regression analysis demonstrating the association of total body weight reduction with changes in fat‐mass.
**Figure S11:** Meta‐regression analysis demonstrating the association of total body weight reduction with changes in triglycerides.
**Figure S12:** Meta‐regression analysis demonstrating the association of total body weight reduction with changes in low‐density lipoprotein (LDL‐C).
**Figure S13:** Meta‐regression analysis demonstrating the association of total body weight reduction with changes in high‐density lipoprotein (HDL‐C).
**Figure S14:** Summary plot of the risk of bias for the included studies, assessed using the Risk of Bias tool 2 (RoB 2).
**Figure S15:**. Summary plot of the risk of bias for the included studies, assessed using the Risk of Bias tool 2 (RoB 2).
**Table S1:** PRISMA NMA Checklist of Items to Include When Reporting A Systematic Review Involving a Network Meta‐analysis.
**Table S2:** A priori defined inclusion and exclusion criteria according to the Population (P), Intervention (I), Comparison (C), Outcomes (O), and Study design (S), (PICOS) framework.
**Table S3:** Search strategy employed for each database.
**Table S4:** Descriptive characteristics of the studies included.
**Table S5:** Ranked interventions by outcome.
**Table S6:** Univariable moderation analyses for body composition and blood outcomes.
**Table S7:** League table with pooled standardized mean di<erence for di<erent interventions and anthropometrics characteristics.
**Table S8:** League table with pooled standardized mean di<erence for di<erent interventions and glycemic profile.
**Table S9:** League table with pooled standardized mean di<erence for di<erent interventions and lipid profile.

## Data Availability

Data sharing not applicable to this article as no datasets were generated or analysed during the current study.
