# Supplementary material for: Comparative Efficacy of GLP‐1 Receptor Agonists, Exercise, and Their Combination on Body Composition and Glucolipid Metabolism in Adults With Overweight or Obesity: A Network Meta‐Analysis of Randomized Controlled Trials
Source: Obes Rev. 2026 Jul 13;27(9):e70189. doi: 10.1111/obr.70189 (PMC13460723; doi:10.1111/obr.70189)
Supplement: Supplementary file 1 [file OBR-27-e70189-s001.pdf]

Comparative efficacy of GLP-1 receptor agonists, exercise, and their combination on body composition and glucolipid metabolism in adults with overweight or obesity: a network meta-analysis of randomized controlled trials

Supplementary materials

Matteo Vandoni[1, †], Alessandro Gatti[1,2, †], Virginia Rossi[3,4], Matteo Giuriato[5], Vittoria Carnevale Pellino[5], Gianvincenzo Zuccotti[3,4,*], Valeria Calcaterra[3,6]

[1] Laboratory of Adapted Motor Activity (LAMA) - Department of Public health, Experimental Medicine and Forensic Science, University of Pavia, 27100 Pavia, Italy

[2] National PhD Programme in One Health approaches to infectious diseases and life science research, Department of Public Health, Experimental and Forensic Medicine, University of Pavia, Pavia, 27100, Italy

[3] Pediatric Department, "Vittore Buzzi" Children's Hospital, 20154 Milan, Italy

[4] Department of Biomedical and Clinical Science, University of Milano, 20157 Milan, Italy

[5] Department of Education and Sport Sciences, Pegaso University, 80143 Naples, Italy

[6] Pediatric and Adolescent Unit, Department of Internal Medicine, University of Pavia, 27100 Pavia, Italy

† These authors contributed equally to this work

Key words: GLP-1, exercise, weight, cardiometabolic, overweight, obesity

Running title: GLP-1 receptor agonists and exercise

*** Corresponding author:**

Gianvincenzo Zuccotti

Department of Biomedical and Clinical Science,

University of Milano

Via GB Grassi n.2

20157 Milan, Italy

E-mail: gianvincenzo.zuccotti@unimi.it

Index

Figure S1. Funnel plot of network meta-analysis depicting the relationship between effect size versus standard error for the effect of different therapies on weight. *4*

Figure S2. Funnel plot of network meta-analysis depicting the relationship between effect size versus standard error for the effect of different therapies on Waist-to-hip-ratio (WtHr). *5*

Figure S3. Funnel plot of network meta-analysis depicting the relationship between effect size versus standard error for the effect of different therapies on fat-mass. *6*

Figure S4. Funnel plot of network meta-analysis depicting the relationship between effect size versus standard error for the effect of different therapies on fasting glucose.. *7*

Figure S5. Funnel plot of network meta-analysis depicting the relationship between effect size versus standard error for the effect of different therapies on homeostatic model assessment of insulin resistance (HOMA-IR). *8*

Figure S6. Funnel plot of network meta-analysis depicting the relationship between effect size versus standard error for the effect of different therapies on triglycerides. *9*

Figure S7. Funnel plot of network meta-analysis depicting the relationship between effect size versus standard error for the effect of different therapies on LDL-C. *10*

Figure S8. Funnel plot of network meta-analysis depicting the relationship between effect size versus standard error for the effect of different therapies on HDL-C. *11*

Figure S9. Meta-regression analysis demonstrating the association of total body weight reduction with changes in waist-to-hip-ratio (WtHr). *12*

Figure S10. Meta-regression analysis demonstrating the association of total body weight reduction with changes in fat-mass. *13*

Figure S11. Meta-regression analysis demonstrating the association of total body weight reduction with changes in triglycerides. *14*

Figure S12. Meta-regression analysis demonstrating the association of total body weight reduction with changes in low-density lipoprotein (LDL-C). *15*

Figure S13. Meta-regression analysis demonstrating the association of total body weight reduction with changes in high-density lipoprotein (HDL-C). *16*

Figure S14. Summary plot of the risk of bias for the included studies, assessed using the Risk of Bias tool 2 (RoB 2). *17*

Figure S15. Summary plot of the risk of bias for the included studies, assessed using the Risk of Bias tool 2 (RoB 2). *18*

Table S1. PRISMA NMA Checklist of Items to Include When Reporting A Systematic Review Involving a Network Meta-analysis *19*

Table S2. A priori defined inclusion and exclusion criteria according to the Population (P), Intervention (I), Comparison (C), Outcomes (O), and Study design (S), (PICOS) framework. *23*

Table S3. Search strategy employed for each database. *24*

Table S4. Descriptive characteristics of the studies included *26*
Table S5. Ranked interventions by outcome *28*
Table S6. Univariable moderation analyses for body composition and blood outcomes *29*
Table S7. League table with pooled standardized mean difference for different interventions and anthropometrics characteristics.. *30*
Table S8. League table with pooled standardized mean difference for different interventions and glycemic profile. *31*
Table S9. League table with pooled standardized mean difference for different interventions and lipid profile. *32*
Extended Methods *33*

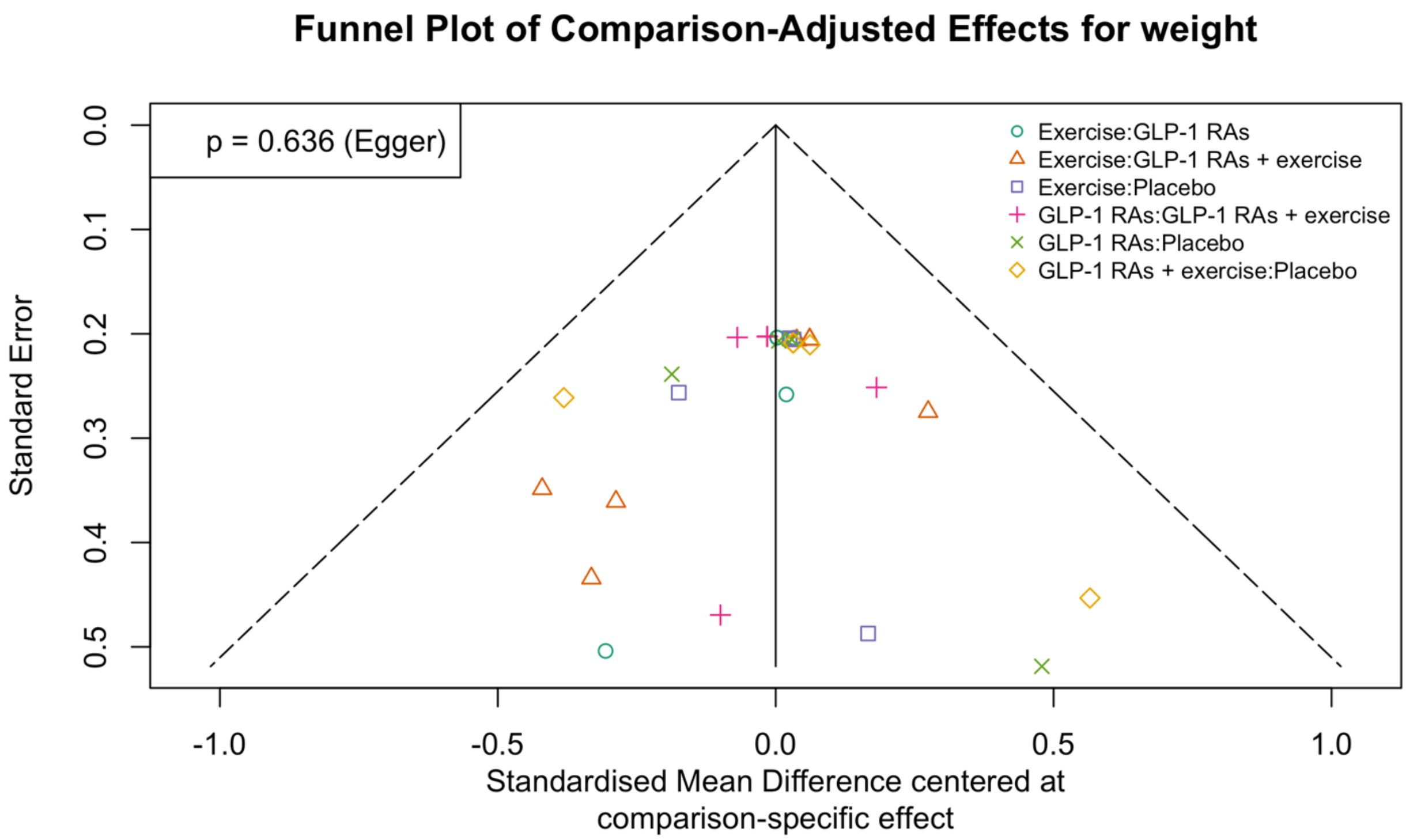

Figure S1. Funnel plot of network meta-analysis depicting the relationship between effect size versus standard error for the effect of different therapies on weight.

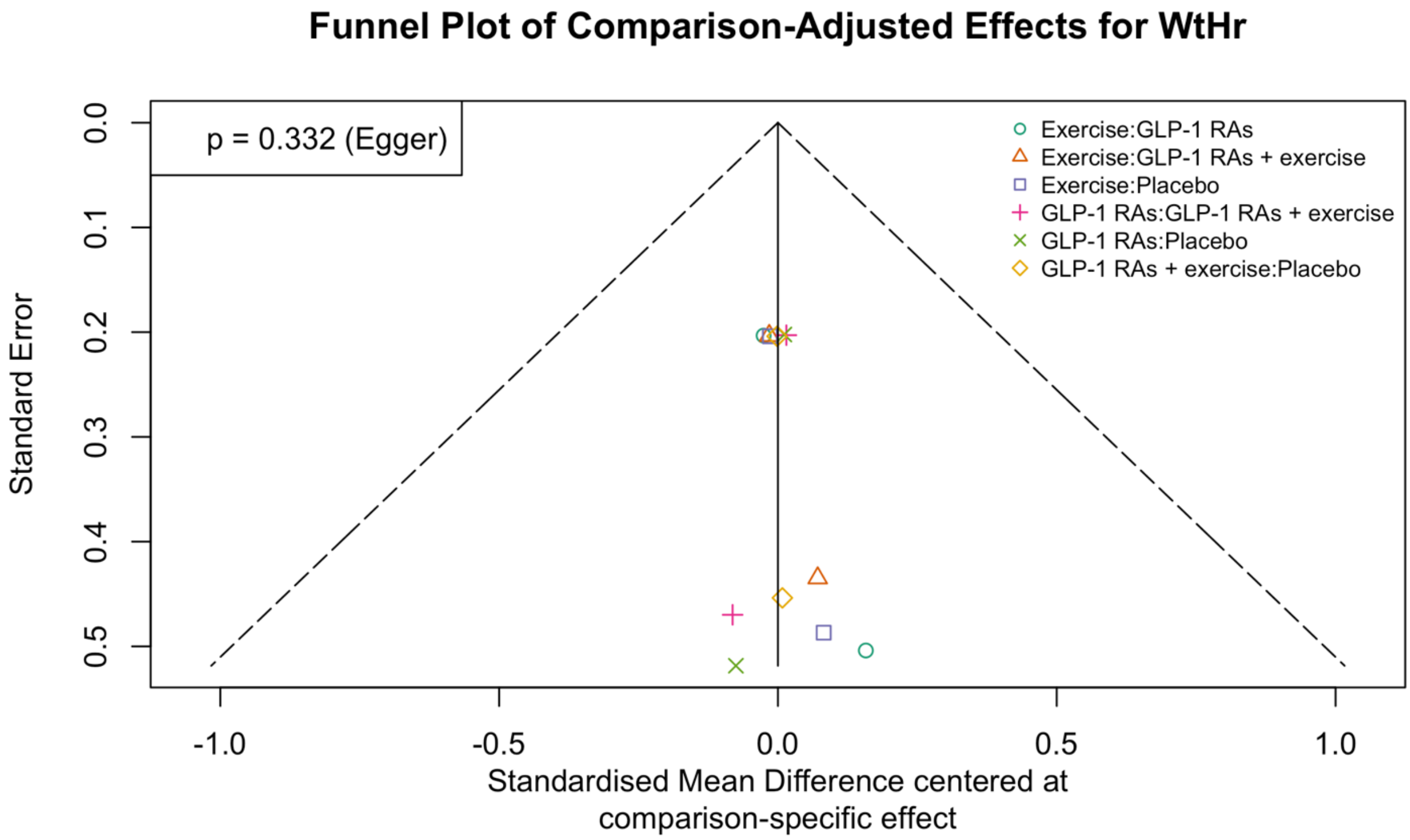

Figure S2. Funnel plot of network meta-analysis depicting the relationship between effect size versus standard error for the effect of different therapies on Waist-to-hip-ratio (WtHr).

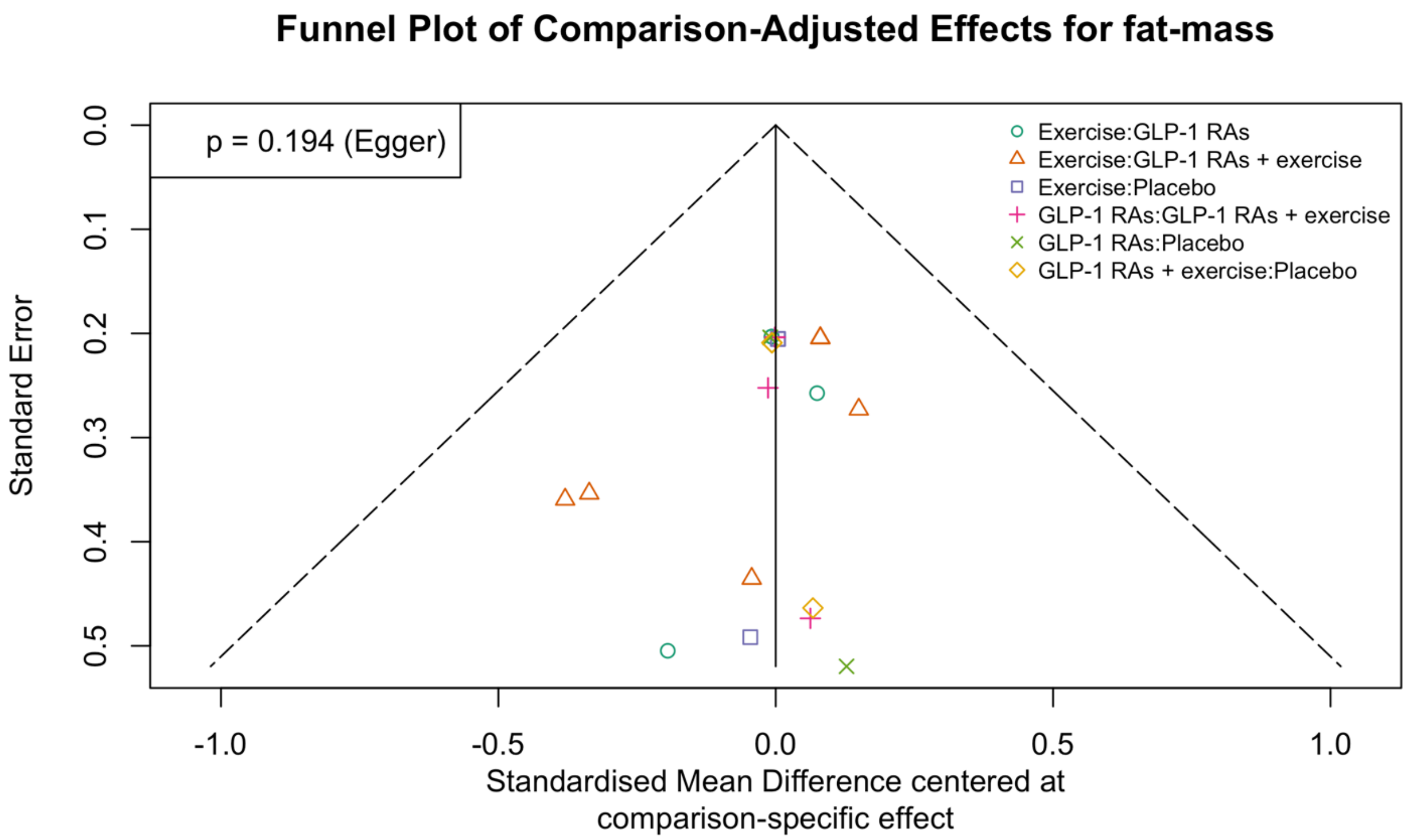

Figure S3. Funnel plot of network meta-analysis depicting the relationship between effect size versus standard error for the effect of different therapies on fat-mass.

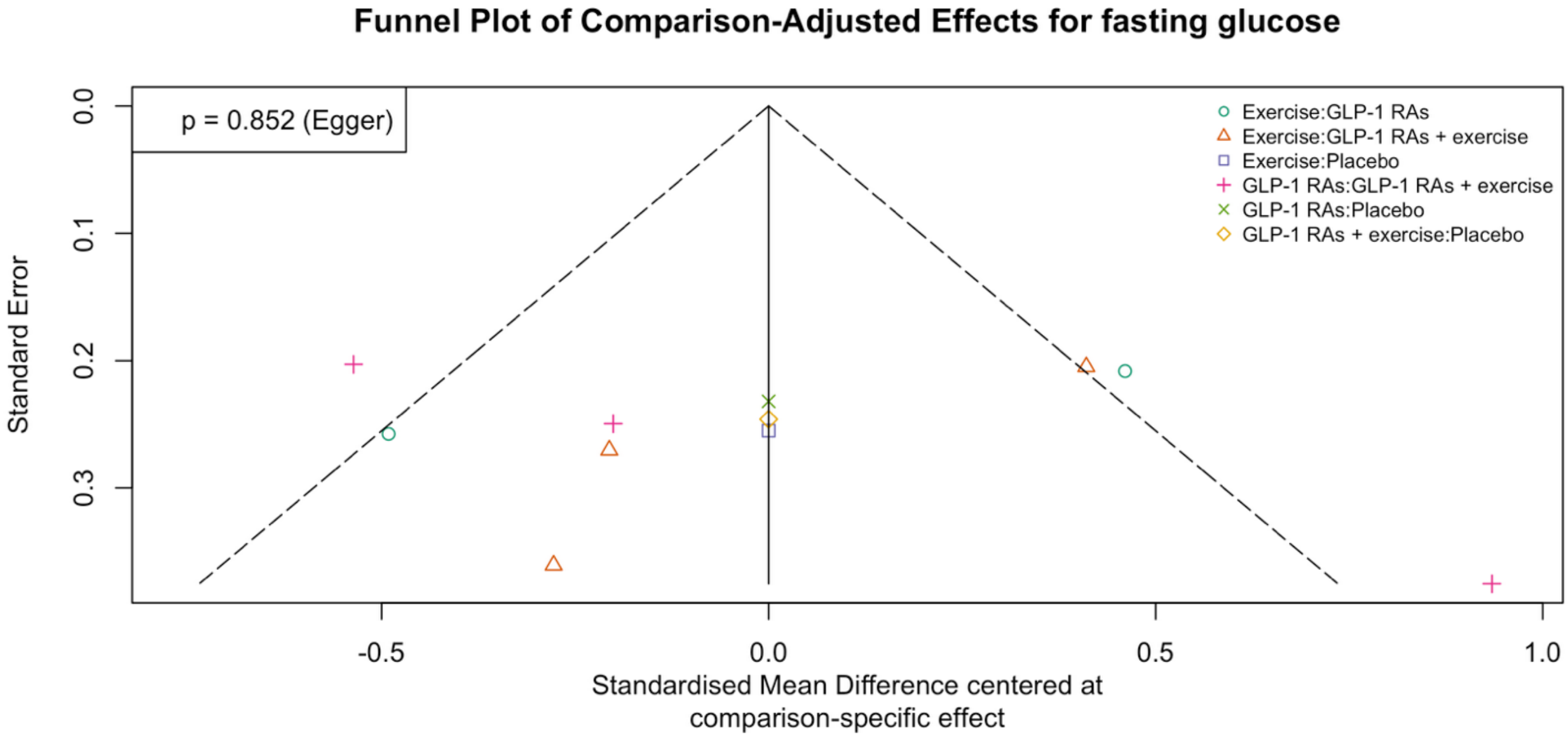

Figure S4. Funnel plot of network meta-analysis depicting the relationship between effect size versus standard error for the effect of different therapies on fasting glucose.

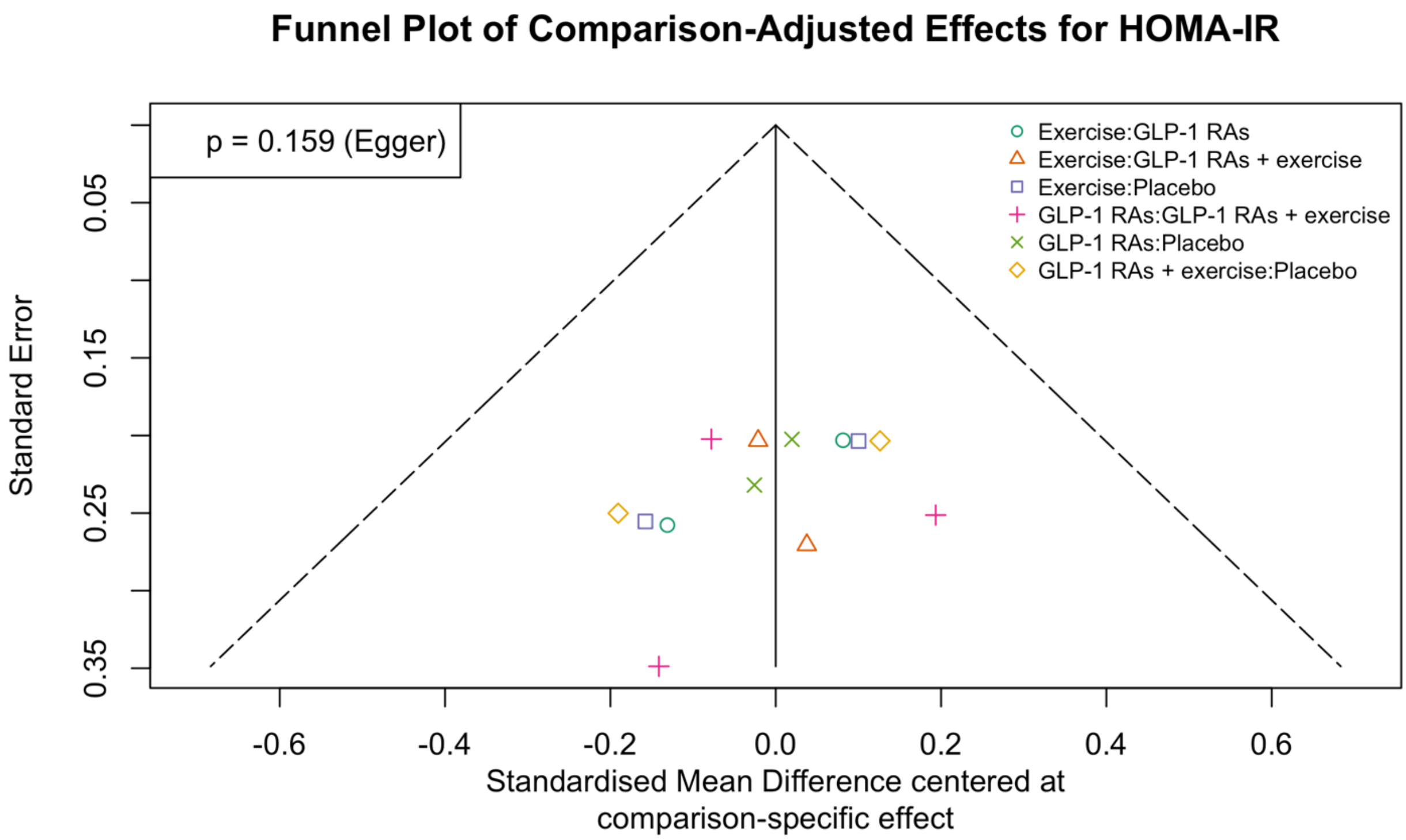

Figure S5. Funnel plot of network meta-analysis depicting the relationship between effect size versus standard error for the effect of different therapies on homeostatic model assessment of insulin resistance (HOMA-IR).

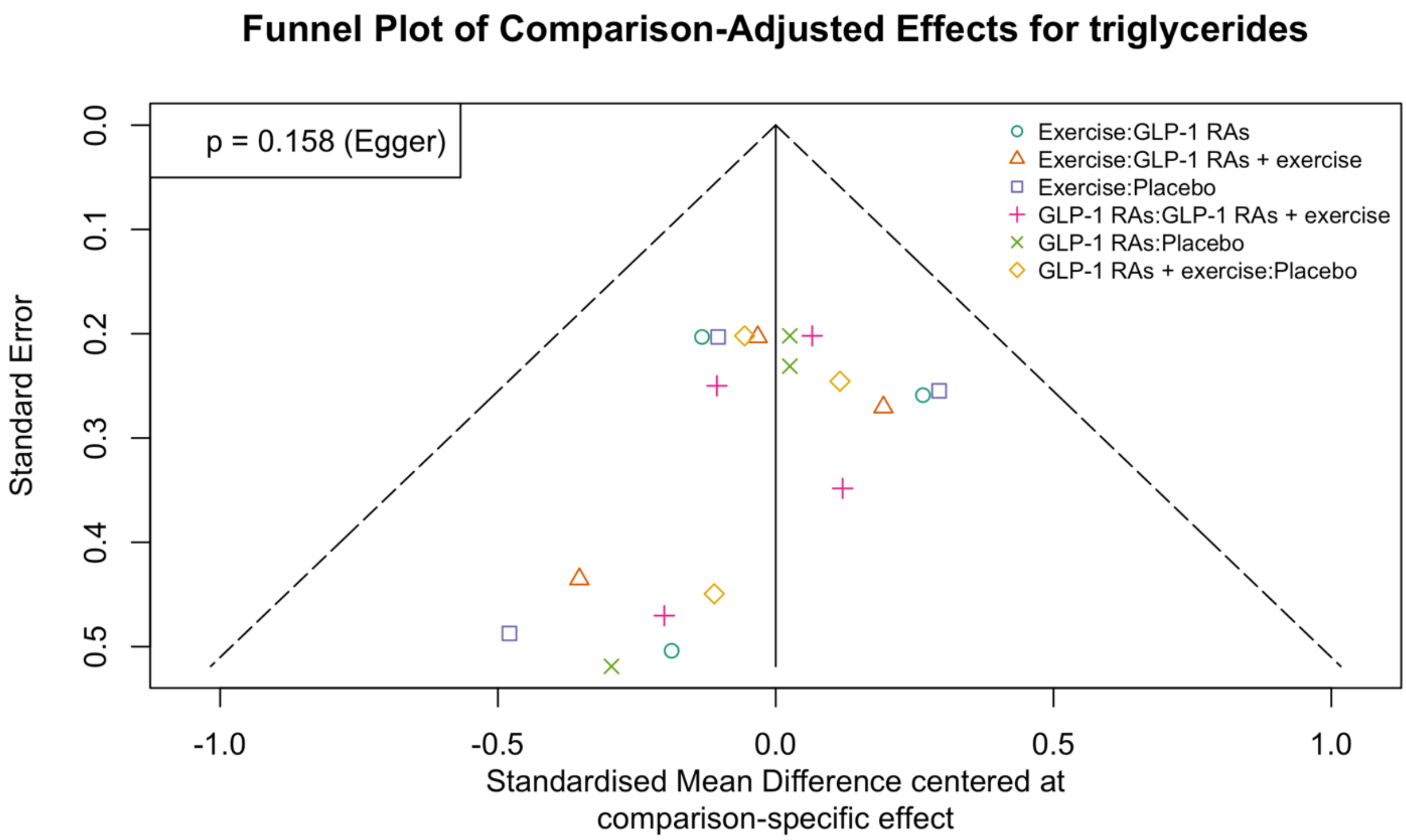

Figure S6. Funnel plot of network meta-analysis depicting the relationship between effect size versus standard error for the effect of different therapies on triglycerides.

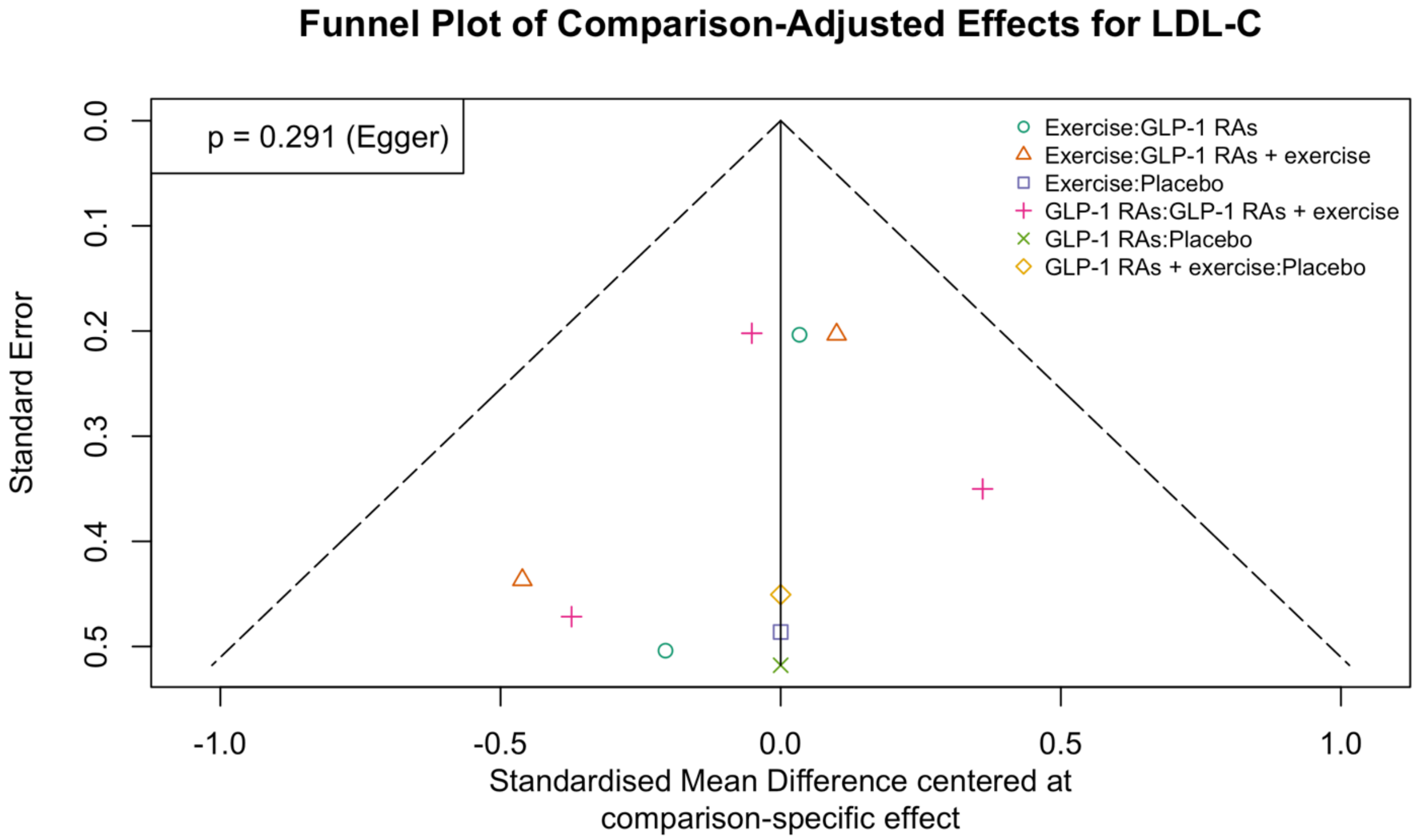

Figure S7. Funnel plot of network meta-analysis depicting the relationship between effect size versus standard error for the effect of different therapies on LDL-C.

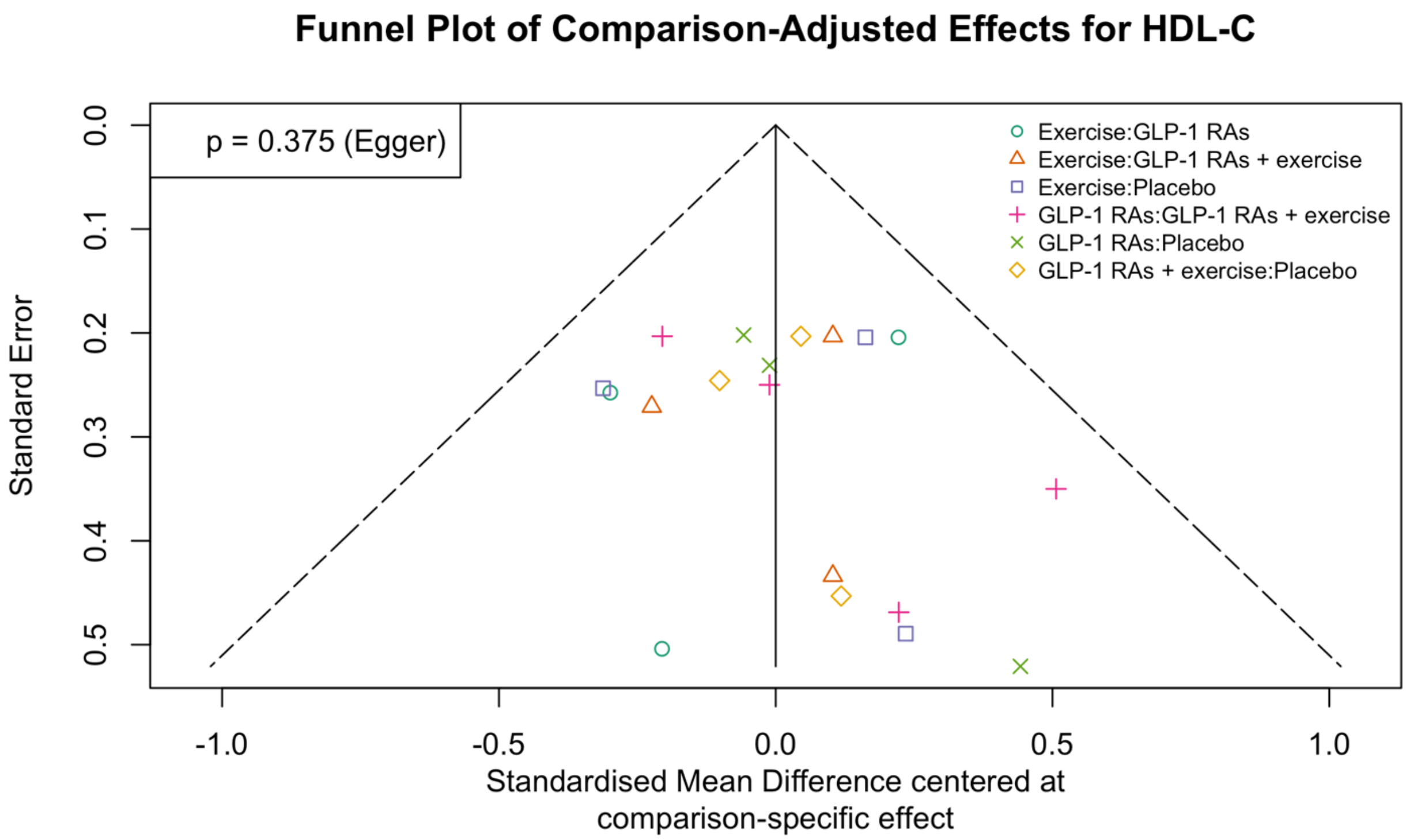

Figure S8. Funnel plot of network meta-analysis depicting the relationship between effect size versus standard error for the effect of different therapies on HDL-C.

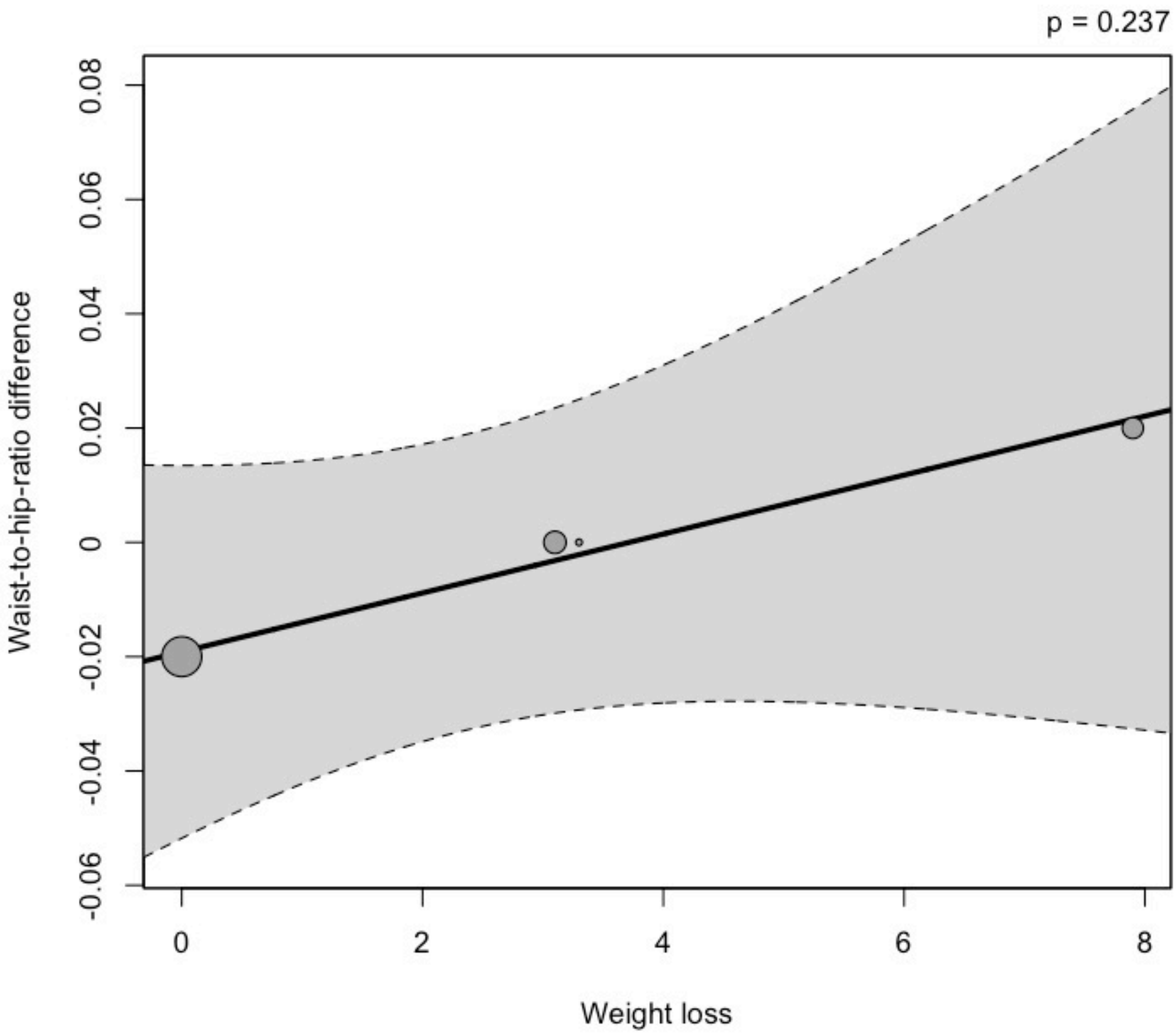

Figure S9. Meta-regression analysis demonstrating the association of total body weight reduction with changes in waist-to-hip-ratio (WtHr).

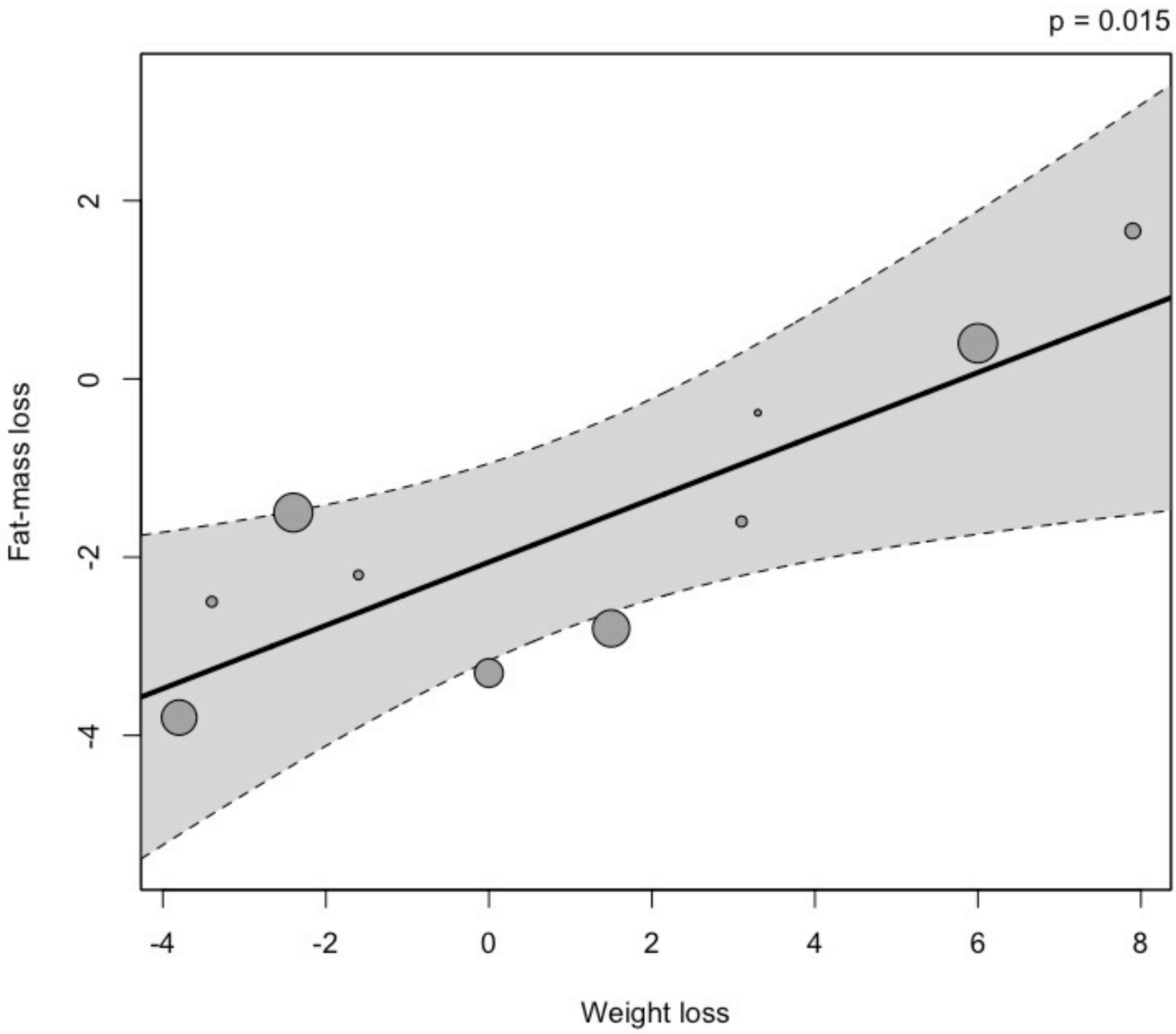

Figure S10. Meta-regression analysis demonstrating the association of total body weight reduction with changes in fat-mass.

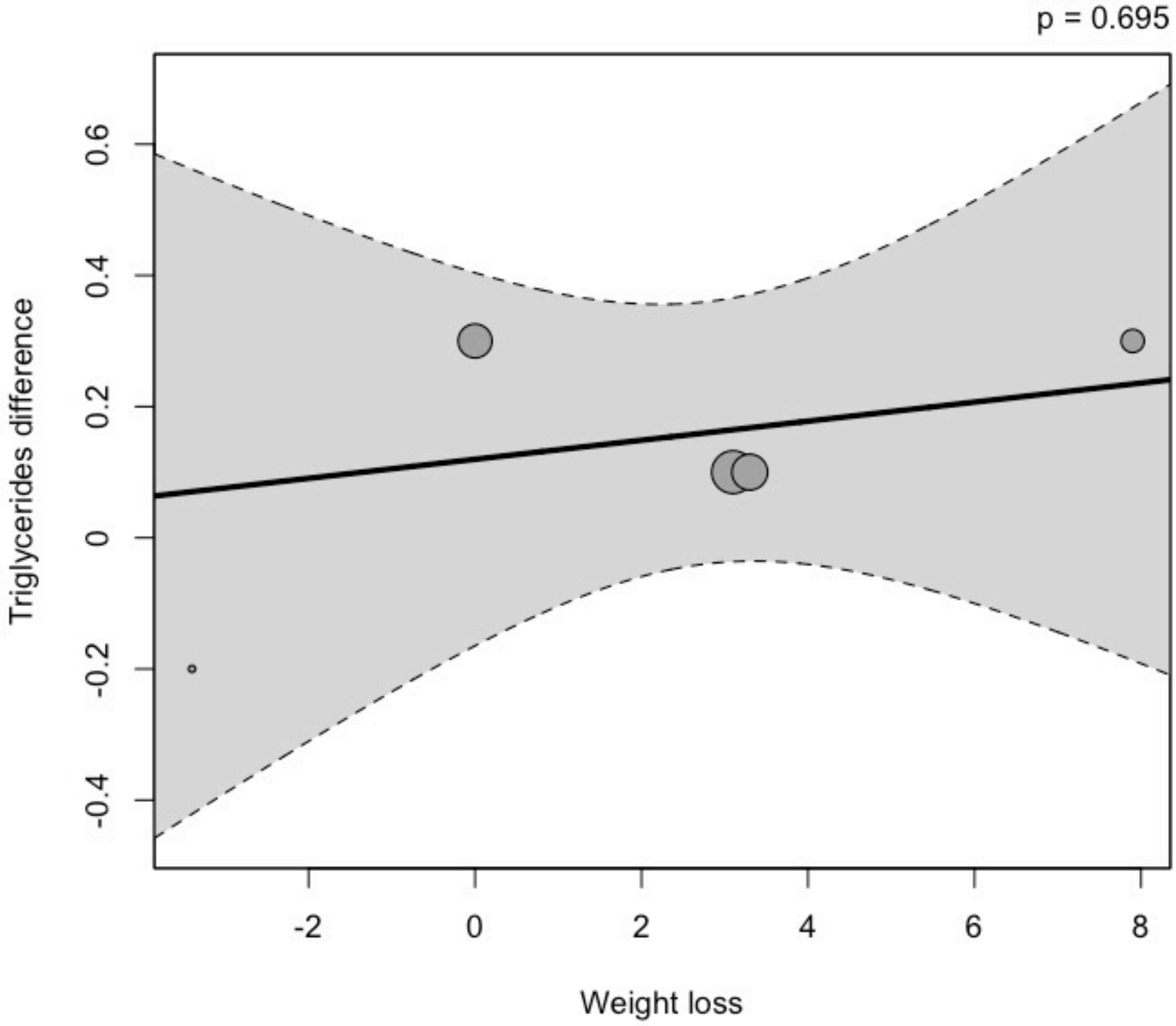

Figure S11. Meta-regression analysis demonstrating the association of total body weight reduction with changes in triglycerides.

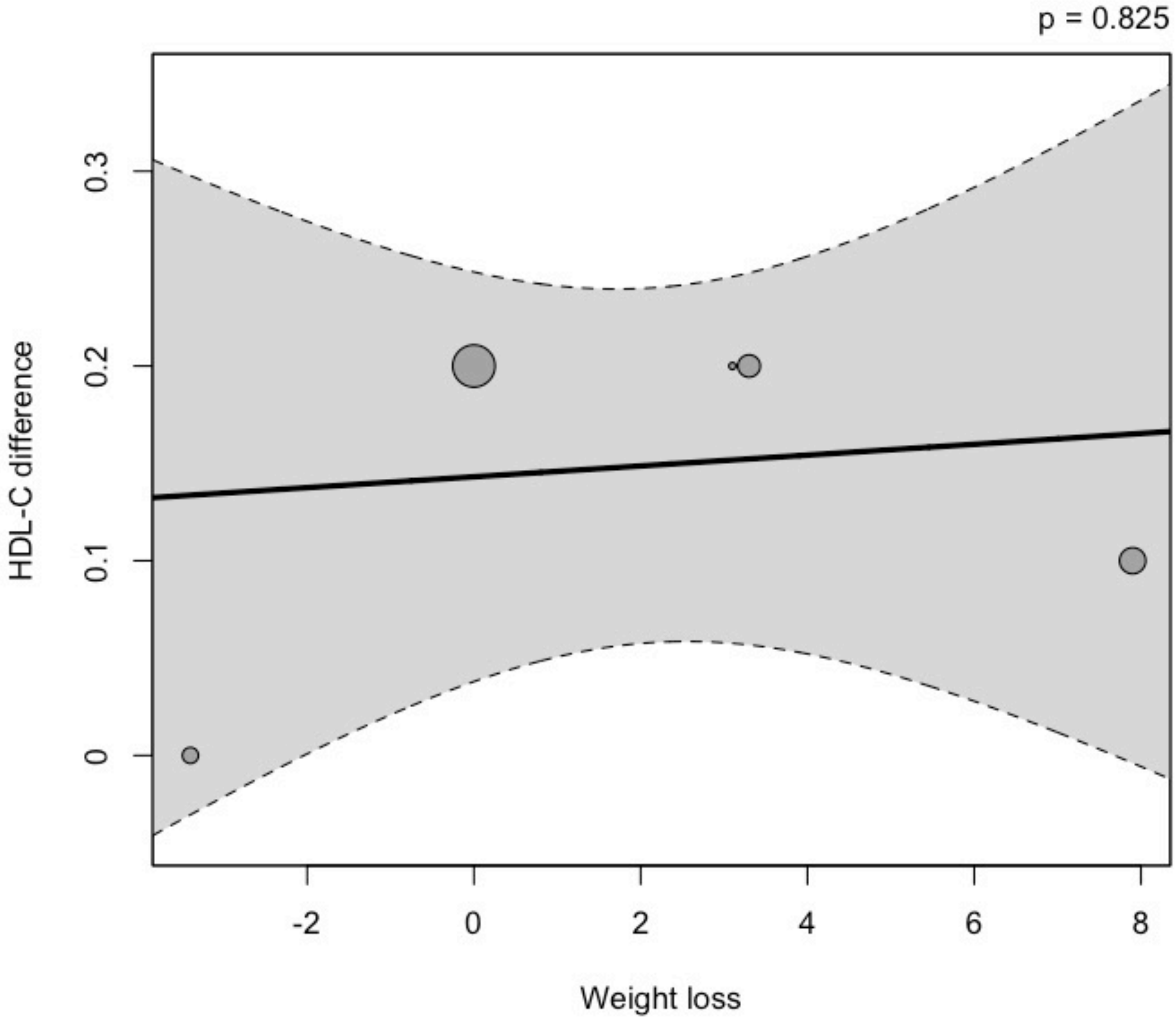

Figure S12. Meta-regression analysis demonstrating the association of total body weight reduction with changes in low-density lipoprotein (LDL-C).

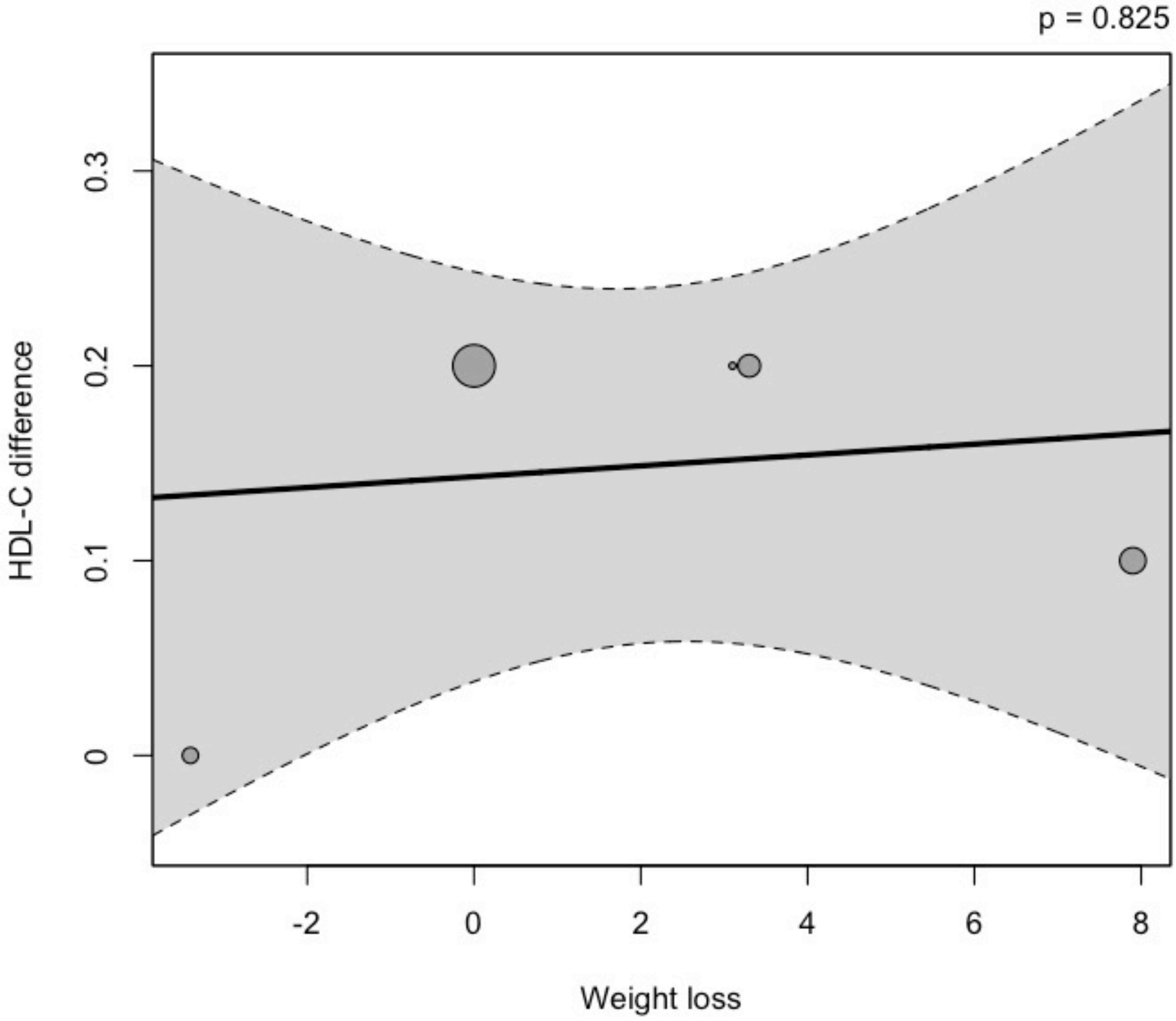

Figure S13. Meta-regression analysis demonstrating the association of total body weight reduction with changes in high-density lipoprotein (HDL-C).

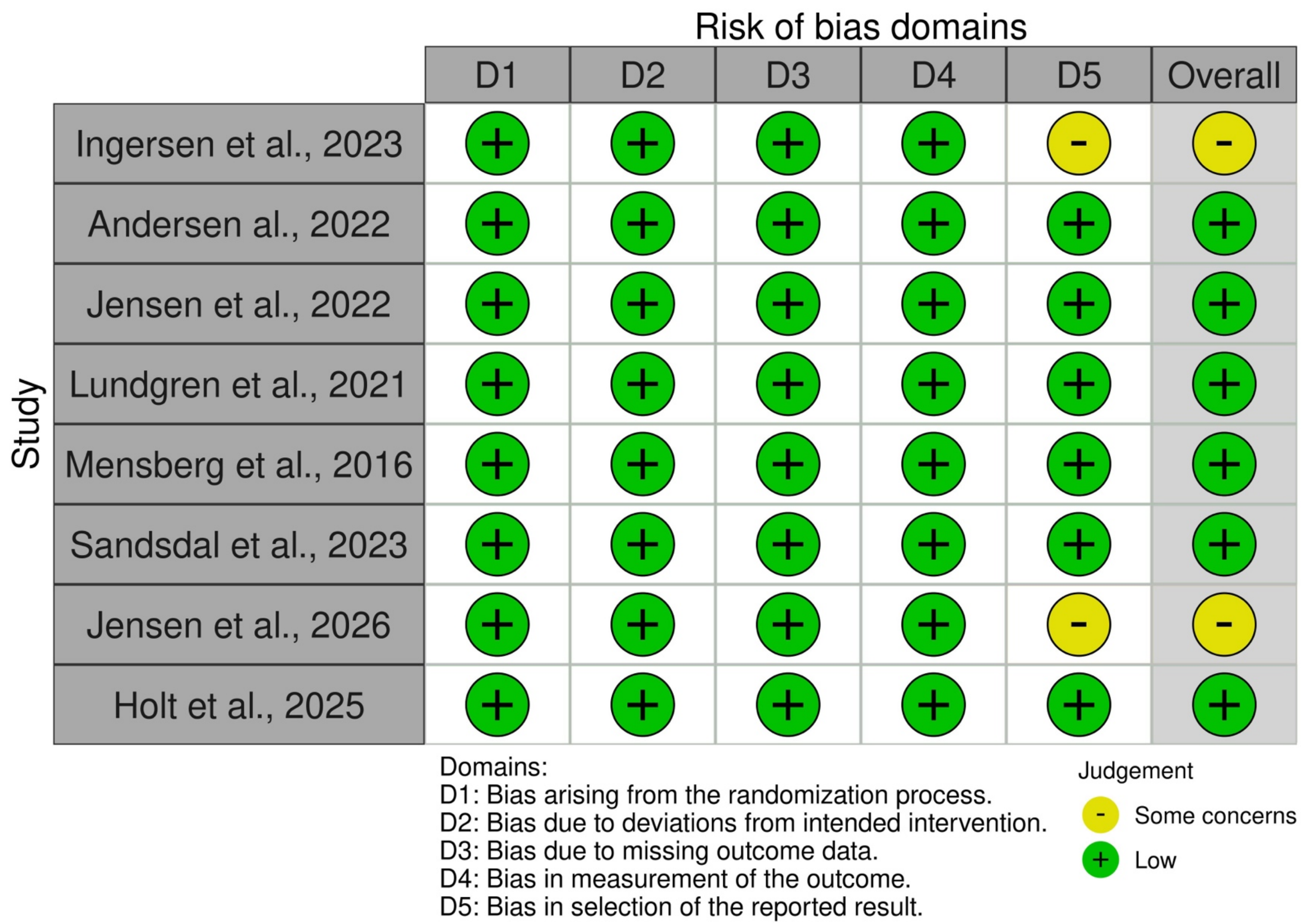

Figure S14. Summary plot of the risk of bias for the included studies, assessed using the Risk of Bias tool 2 (RoB 2).

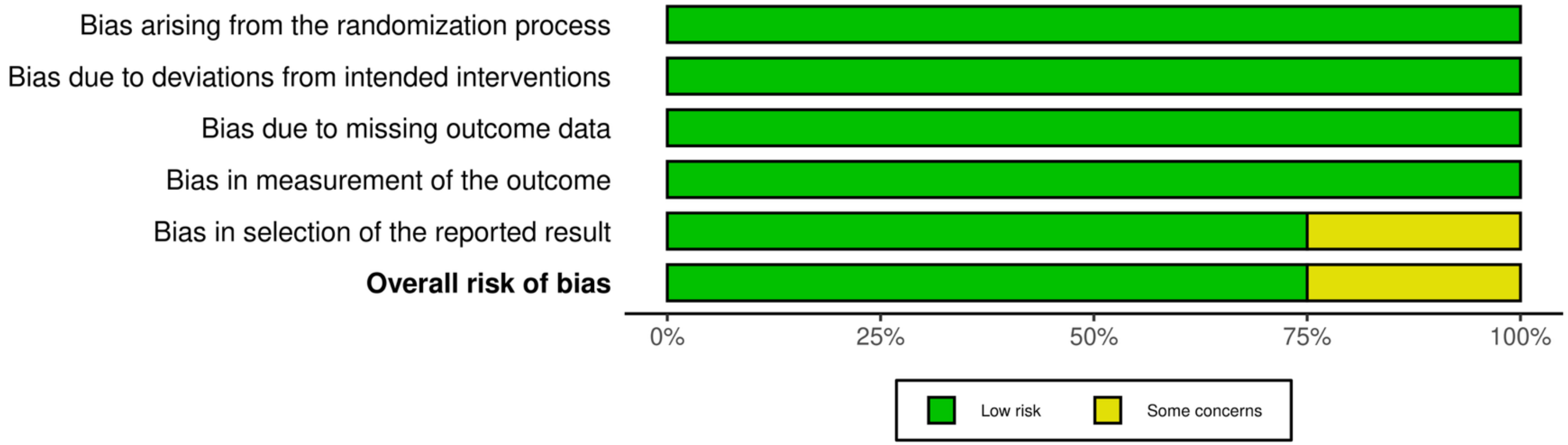

Figure S15. Summary plot of the risk of bias for the included studies, assessed using the Risk of Bias tool 2 (RoB 2).

Table S1. PRISMA NMA Checklist of Items to Include When Reporting A Systematic Review Involving a Network Meta-analysis

Section/Topic	Item #	Checklist Item	Reported on Page #
**TITLE**			
Title	1	Identify the report as a systematic review *incorporating a network meta-analysis (or related form of meta-analysis).*	1
**ABSTRACT**			
Structured summary	2	Provide a structured summary including, as applicable:<br>**Background:** main objectives<br>**Methods:** data sources; study eligibility criteria, participants, and interventions; study appraisal; and *synthesis methods, such as network meta-analysis.*<br>**Results:** number of studies and participants identified; summary estimates with corresponding confidence/credible intervals; *treatment rankings may also be discussed. Authors may choose to summarize pairwise comparisons against a chosen treatment included in their analyses for brevity.*<br>**Discussion/Conclusions:** limitations; conclusions and implications of findings.<br>**Other:** primary source of funding; systematic review registration number with registry name.	1-2
**INTRODUCTION**			
Rationale	3	Describe the rationale for the review in the context of what is already known*, including mention of why a network meta-analysis has been conducted.*	3
Objectives	4	Provide an explicit statement of questions being addressed, with reference to participants, interventions, comparisons, outcomes, and study design (PICOS).	3-4
**METHODS**			
Protocol and registration	5	Indicate whether a review protocol exists and if and where it can be accessed (e.g., Web address); and, if available, provide registration information, including registration number.	5
Eligibility criteria	6	Specify study characteristics (e.g., PICOS, length of follow-up) and report characteristics (e.g., years considered, language, publication status) used as criteria for eligibility, giving rationale. *Clearly describe eligible treatments included in the treatment network, and note whether any have been clustered or merged into the same node (with justification).*	5; Supp.
Information sources	7	Describe all information sources (e.g., databases with dates of coverage, contact with study authors to identify additional studies) in the search and date last searched.	5; Supp.
Search	8	Present full electronic search strategy for at least one database, including any limits used, such that it could be repeated.	5; Supp.
Study selection	9	State the process for selecting studies (i.e., screening, eligibility, included in systematic review, and, if applicable, included in the meta-analysis).	5-6

Data collection process	10	Describe method of data extraction from reports (e.g., piloted forms, independently, in duplicate) and any processes for obtaining and confirming data from investigators.	6
Data items	11	List and define all variables for which data were sought (e.g., PICOS, funding sources) and any assumptions and simplifications made.	6; Supp.
**Geometry of the network**	**S1**	Describe methods used to explore the geometry of the treatment network under study and potential biases related to it. This should include how the evidence base has been graphically summarized for presentation, and what characteristics were compiled and used to describe the evidence base to readers.	6-7
Risk of bias within individual studies	12	Describe methods used for assessing risk of bias of individual studies (including specification of whether this was done at the study or outcome level), and how this information is to be used in any data synthesis.	6
Summary measures	13	State the principal summary measures (e.g., risk ratio, difference in means). *Also describe the use of additional summary measures assessed, such as treatment rankings and surface under the cumulative ranking curve (SUCRA) values, as well as modified approaches used to present summary findings from meta-analyses.*	6-7
Planned methods of analysis	14	Describe the methods of handling data and combining results of studies for each network meta-analysis. This should include, but not be limited to:<br>• *Handling of multi-arm trials;*<br>• *Selection of variance structure;*<br>• *Selection of prior distributions in Bayesian analyses; and*<br>• *Assessment of model fit.*	6-7
**Assessment of Inconsistency**	**S2**	Describe the statistical methods used to evaluate the agreement of direct and indirect evidence in the treatment network(s) studied. Describe efforts taken to address its presence when found.	6-7
Risk of bias across studies	15	Specify any assessment of risk of bias that may affect the cumulative evidence (e.g., publication bias, selective reporting within studies).	6-7
Additional analyses	16	Describe methods of additional analyses if done, indicating which were pre-specified. This may include, but not be limited to, the following:<br>• Sensitivity or subgroup analyses;<br>• Meta-regression analyses;<br>• *Alternative formulations of the treatment network; and*<br>• *Use of alternative prior distributions for Bayesian analyses (if applicable).*	6-7
**RESULTS†**			
Study selection	17	Give numbers of studies screened, assessed for eligibility, and included in the review, with reasons for exclusions at each stage, ideally with a flow diagram.	8
**Presentation of**	**S3**	Provide a network graph of the included studies to enable	8

**network structure**		visualization of the geometry of the treatment network.	
**Summary of network geometry**	**S4**	Provide a brief overview of characteristics of the treatment network. This may include commentary on the abundance of trials and randomized patients for the different interventions and pairwise comparisons in the network, gaps of evidence in the treatment network, and potential biases reflected by the network structure.	8
Study characteristics	18	For each study, present characteristics for which data were extracted (e.g., study size, PICOS, follow-up period) and provide the citations.	Supp.
Risk of bias within studies	19	Present data on risk of bias of each study and, if available, any outcome level assessment.	8, Supp.
Results of individual studies	20	For all outcomes considered (benefits or harms), present, for each study: 1) simple summary data for each intervention group, and 2) effect estimates and confidence intervals. *Modified approaches may be needed to deal with information from larger networks.*	Supp.
Synthesis of results	21	Present results of each meta-analysis done, including confidence/credible intervals. *In larger networks, authors may focus on comparisons versus a particular comparator (e.g. placebo or standard care), with full findings presented in an appendix. League tables and forest plots may be considered to summarize pairwise comparisons.* If additional summary measures were explored (such as treatment rankings), these should also be presented.	8-10; Supp.
**Exploration for inconsistency**	**S5**	Describe results from investigations of inconsistency. This may include such information as measures of model fit to compare consistency and inconsistency models, *P* values from statistical tests, or summary of inconsistency estimates from different parts of the treatment network.	Supp.
Risk of bias across studies	22	Present results of any assessment of risk of bias across studies for the evidence base being studied.	Supp.
Results of additional analyses	23	Give results of additional analyses, if done (e.g., sensitivity or subgroup analyses, meta-regression analyses*, alternative network geometries studied, alternative choice of prior distributions for Bayesian analyses,* and so forth).	Supp.
**DISCUSSION**			
Summary of evidence	24	Summarize the main findings, including the strength of evidence for each main outcome; consider their relevance to key groups (e.g., healthcare providers, users, and policy-makers).	11
Limitations	25	Discuss limitations at study and outcome level (e.g., risk of bias), and at review level (e.g., incomplete retrieval of identified research, reporting bias). *Comment on the validity of the assumptions, such as transitivity and consistency. Comment on any concerns regarding network geometry (e.g., avoidance of certain comparisons).*	13
Conclusions	26	Provide a general interpretation of the results in the context of other evidence, and implications for future research.	12-13
**FUNDING**			
Funding	27	Describe sources of funding for the systematic review and other support (e.g., supply of data); role of funders for the systematic	Not applicable

		review. This should also include information regarding whether funding has been received from manufacturers of treatments in the network and/or whether some of the authors are content experts with professional conflicts of interest that could affect use of treatments in the network.	

PICOS = population, intervention, comparators, outcomes, study design.

Table S2. *A priori* defined inclusion and exclusion criteria according to the Population (P), Intervention (I), Comparison (C), Outcomes (O), and Study design (S), (PICOS) framework.

**Search Strategy**	**Details**
Inclusion Criteria	P: Adults aged 18–65 years with overweight or obesity (BMI ≥ 25 kg/m²) with or without type 2 diabetes and without any psychiatric, cognitive, or other non-communicable/acute diseases
	I: GLP-1 receptor agonists and structured exercise combined
	C: Placebo, GLP-1 RAs alone, or exercise alone
	O: body composition, metabolic biomarkers, cardiovascular biomarkers
	S: original data (randomized controlled trial)
Exclusion Criteria	P: adults with overweight and obesity presenting mental or physical health disorders, physical limitations that prevent physical participation, people who underwent recent bariatric surgery; older adults with overweight/obesity
	I: GLP-1 receptor agonists + physical activity intervention (advising participant only to do physical activities following WHO guidelines)
	S: qualitative studies, non-original data (opinion papers, review articles, letters, protocols, commentaries), non RCT-clinical trials
Language	English
Time filter	Studies published from the 1990
Database	PubMed/MEDLINE; Web of Science; Scopus; EBSCO

Table S3. Search strategy employed for each database.

Database	Search Terms	Date Range
**Pubmed/Medline**	(Obesity[Mesh] OR obes*[tiab] OR overweight[tiab])<br>AND<br>(Exercise[Mesh] OR exercise[tiab] OR exercise[Mesh] OR "physical activity"[tiab] OR training[tiab] OR aerobic[tiab] OR "resistance training"[tiab] OR fitness[tiab])<br>AND<br>("Anti-Obesity Agents” [MeSH] OR "Glucagon-Like Peptide-1 Receptor"[MeSH] OR GLP-1[tiab] OR "GLP-1 drug*"[tiab] OR GLP1[tiab] OR "GLP-1 receptor agonist"[tiab] OR "GLP-1 receptor*"[tiab] OR liraglutide[tiab] OR semaglutide[tiab] OR exenatide[tiab] OR dulaglutide[tiab] OR lixisenatide[tiab] OR albiglutide[tiab])<br>AND<br>(weight*[tiab] OR "fat mass"[tiab] OR "lean mass"[tiab] OR "body mass"[tiab] OR "body composition"[tiab] OR BMI[tiab] OR "waist circumference"[tiab] OR cardiometabolic[tiab] OR glucose[tiab] OR "insulin resistance"[tiab] OR lipid[tiab] OR cholesterol[tiab] OR "blood pressure"[tiab])	1990-01-01 to 2026-04-30
**Web of Science**	(Obesity OR obes* OR overweight)<br>AND<br>(Exercise OR exercise OR exercise OR "physical activity" OR training OR aerobic OR "resistance training" OR fitness)<br>AND<br>("Anti-Obesity Agents[MeSH Terms]" OR "Glucagon-Like Peptide-1 Receptor Agonists[MeSH Terms]" OR GLP-1 OR "GLP-1 drug*" OR GLP1 OR "GLP-1 receptor agonist" OR "GLP-1 receptor*" OR liraglutide OR semaglutide OR exenatide OR dulaglutide OR lixisenatide OR albiglutide)<br>AND<br>(weight* OR "fat mass" OR "lean mass" OR "body mass" OR "body composition" OR BMI OR "waist circumference" OR cardiometabolic OR glucose OR "insulin resistance" OR lipid OR cholesterol OR "blood pressure")	1990-01-01 to 2026-04-30
**Scopus**	(INDEXTERMS(obesity) OR TITLE-ABS(obes*) OR TITLE-ABS(overweight))<br>AND<br>(INDEXTERMS(exercise) OR TITLE-ABS(exercise) OR TITLE-ABS("physical activity") OR TITLE-ABS(training) OR TITLE-ABS(aerobic) OR TITLE-ABS("resistance training") OR TITLE-ABS(fitness))<br>AND<br>(TITLE-ABS("Anti-Obesity Agents") OR TITLE-ABS("Glucagon-Like Peptide-1 Receptor Agonists") OR TITLE-ABS(glp-1) OR TITLE-ABS(glp-1*) OR TITLE-ABS(glp1) OR TITLE-ABS("GLP-1 receptor agonist") OR TITLE-ABS("GLP-1 receptor*") OR TITLE-ABS(liraglutide) OR TITLE-ABS(semaglutide) OR TITLE-ABS(exenatide) OR TITLE-ABS(dulaglutide) OR TITLE-ABS(lixisenatide) OR TITLE-ABS(albiglutide))<br>AND<br>(TITLE-ABS(weight*) OR TITLE-ABS("fat mass") OR TITLE-ABS("lean mass") OR TITLE-ABS("body mass") OR TITLE-ABS("body composition") OR TITLE-ABS(bmi) OR TITLE-ABS("waist circumference") OR TITLE-ABS(cardiometabolic) OR TITLE-ABS(glucose) OR TITLE-ABS("insulin resistance") OR TITLE-ABS(lipid) OR TITLE-ABS(cholesterol) OR TITLE-ABS("blood pressure"))<br>AND<br>(LIMIT-TO(DOCTYPE, "ar"))<br>AND<br>(LIMIT-TO(LANGUAGE, "English"))	1990-01-01 to 2026-04-30

**EBSCO**	(MH "Obesity" OR TX obes* OR TX overweight)<br>AND<br>(MH "Exercise" OR TX exercise OR TX "physical activity" OR TX training OR TX aerobic OR TX "resistance training" OR TX fitness)<br>AND<br>(MH "Anti-Obesity Agents" OR MH "Glucagon-Like Peptide-1 Receptor" OR TX GLP-1 OR TX "GLP-1 drug*" OR TX GLP1 OR TX "GLP-1 receptor agonist" OR TX "GLP-1 receptor*" OR TX liraglutide OR TX semaglutide OR TX exenatide OR TX dulaglutide OR TX lixisenatide OR TX albiglutide)<br>AND<br>(TX weight* OR TX "fat mass" OR TX "lean mass" OR TX "body mass" OR TX "body composition" OR TX BMI OR TX "waist circumference" OR TX cardiometabolic OR TX glucose OR TX "insulin resistance" OR TX lipid OR TX cholesterol OR TX "blood pressure")	1990-01-01 to 2026-04-30

Table S4. Descriptive characteristics of the studies included

	Group	Sample size	Age (years)	Weight (kg)	BMI (kg/m^2)	Type of training	Intervention duration (weeks)	Outcomes
**Lundgren et al., 2021**	GLP-1 RAs + exercise	18 (M) 31 (W)	42 ± 12	98.3 ± 11.5	32.8 ± 2.4	Combined training + Liraglutide 3.0mg/day	52	Body composition, glycemic and lipid profiles, physical function, emotional well-being.
	GLP-1 RAs	18 (M) 31 (W)	43 ± 12	95.1 ± 12.8	32.7 ± 3.1	Liraglutide 3.0mg/day		
	Exercise	17 (M) 31 (W)	43 ± 12	96.8 ± 13.2	32.7 ± 3.0	Combined training + placebo		
	Placebo	18 (M) 31 (W)	43 ± 12	96.7 ± 12.7	32.3 ± 3.0	Placebo		
**Sandsdal et al., 2023 (CIT)**	Placebo	15 (M) 24 (W)	43.7 ± 12.3	96.6 ± 13.2	32.1 ± 2.9	Placebo	52	Body composition, glycemic and lipid profiles, cardiometabolic risk, inflammatory markers
	Exercise	11 (M) 15 (W)	45.0 ± 11.8	96.1 ± 13.2	32.2 ± 3.3	Combined training + placebo		
	GLP-1 RAs	13 (M) 23 (W)	46.2 ± 10.3	94.8 ± 11.3	32.5 ± 3.1	Liraglutide 3.0mg/day		
	GLP-1 RAs + exercise	11 (M) 18 (W)	44.5 ± 12.7	99.1 ± 11.4	32.6 ± 2.5	Combined training + Liraglutide 3.0mg/day		
**Jensen et al., 2026**	GLP-1 RAs + exercise	17 (M) 31 (W)	42.3 ± 12.4	98.1 ± 11.5	32.8 ± 2.5	Combined training + Liraglutide 3.0mg/day	52	Body composition, physical fitness
	GLP-1 RAs	18 (M) 31 (W)	43.2 ± 11.6	95.1 ± 12.8	32.3 ± 3.1	Liraglutide 3.0mg/day		
	Exercise	17 (M) 31 (W)	43.0 ± 12.4	96.8 ± 13.2	32.7 ± 3.0	Combined training + placebo		
	Placebo	18 (M) 30 (W)	42.8 ± 11.6	96.9 ± 12.7	32.3 ± 3.0	Placebo		
**Ingersen et al., 2023**	GLP-1 RAs + exercise	13 (M) 2 (W)	56 ± 6	110.3 ± 21.1	34.6 ± 5.4	Aerobic training + Semaglutide 1.0mg/week	12	Body composition, lipid and glycemic profiles, cardiorespiratory fitness
	Exercise	14 (M) 2 (W)	59 ± 6	111.7 ± 22.3	34.8 ± 4.8	Aerobic training		

**Mensberg et al., 2016**	GLP-1 RAs + exercise	13 (M) 4 (W)	56.5 ± 9.0	101.0 ± 14.5	32.5 ± 3.7	Combined training + Liraglutide 1.8mg/week	16	Body composition, lipid and glycemic profiles, physical function
	Exercise	10 (M) 6 (W)	55.6 ± 12.0	97.6 ± 14.9	31.3 ± 3.4	Combined training + placebo		
**Andersen et al., 2022**	GLP-1 RAs + exercise	13 (M)	43 ± 12	104.4 ± 7.61	31.6 ± 3.0	Combined training + Liraglutide 3.0mg/day	52	Body composition, lipid and glycemic profiles, reproductive profile
	Exercise	9 (M)	43 ± 12	101.7 ± 16.9	31.8 ± 3.7	Liraglutide 3.0mg/day		
	GLP-1 RAs	7 (M)	43 ± 12	110.3 ± 12.9	32.4 ± 2.8	Combined training + placebo		
	Placebo	8 (M)	43 ± 12	106.6 ± 12.5	30.8 ± 3.0	Placebo		
**Jensen et al., 2023**	GLP-1 RAs + exercise	71 (M) 124 (W)	43 ± 12	Not reported	32.6 ± 2.9	Combined training + Liraglutide 3.0mg/day	52	Glycemic profile
	Exercise					Liraglutide 3.0mg/day		
	GLP-1 RAs					Combined training + placebo		
	Placebo					Placebo		
**Holt et al., 2025**	Placebo	71 (M) 124 (W)	42 ± 12	96.7 ± 12.7	32.6 ± 2.9	Combined training + Liraglutide 3.0mg/day	52	Body composition, postprandial GLP-1 secretion
	Exercise			96.8 ± 13.2		Liraglutide 3.0mg/day		
	GLP-1 RAs			95.1 ± 12.8		Combined training + placebo		
	GLP-1 RAs + exercise			98.3 ± 11.5		Placebo		

Table S5. Ranked interventions by outcome

Outcome	Rank 1	Rank 2	Rank 3	Rank 4
**Weight**	GLP-1 RAs + exercise (99.9%)	GLP-1 RAs (66.6%)	Exercise (33.5%)	Placebo (0.0%)
**WHtR**	GLP-1 RAs + exercise (94.4%)	Exercise (63.3%)	GLP-1 RAs (36.3%)	Placebo (6.0%)
**Fat mass**	GLP-1 RAs + exercise (100.0%)	Exercise (59.1%)	GLP-1 RAs (40.9%)	Placebo (0.0%)
**Fasting glucose**	GLP-1 RAs + exercise (72.6%)	GLP-1 RAs (54.9%)	Exercise (48.4%)	Placebo (24.2%)
**HOMA-IR**	GLP-1 RAs + exercise (96.1%)	Exercise (59.1%)	GLP-1 RAs (43.9%)	Placebo (1.0%)
**Triglycerides**	GLP-1 RAs (74.5%)	Placebo (69.1%)	GLP-1 RAs + exercise (33.8%)	Exercise (22.6%)
**LDL-C**	GLP-1 RAs (66.8%)	Placebo (54.1%)	GLP-1 RAs + exercise (47.4%)	Exercise (31.7%)
**HDL-C**	GLP-1 RAs + exercise (86.0%)	Exercise (73.2%)	GLP-1 RAs (30.9%)	Placebo (9.9%)

Table S6. Univariable moderation analyses for body composition and blood outcomes

Outcome	Moderator	Unit / comparison	SMD β (95% CI)	p
Weight	Diabetes status	Diabetes vs no diabetes	**0.42 (0.12, 0.72)**	**.021**
Weight	Intervention duration	Per 10 weeks	**-0.11 (-0.21, -0.02)**	**.034**
Weight	Women in sample	Per 10 percentage points	**-0.07 (-0.13, -0.01)**	**.031**
Fat mass (%)	Diabetes status	Diabetes vs no diabetes	**0.44 (0.28, 0.60)**	**.007**
Fat mass (%)	Intervention duration	Per 10 weeks	**-0.12 (-0.14, -0.09)**	**.003**
Fat mass (%)	Women in sample	Per 10 percentage points	-0.04 (-0.23, 0.15)	.485
Waist-to-height ratio	Diabetes status		Not estimable	
Waist-to-height ratio	Intervention duration		Not estimable	
Waist-to-height ratio	Women in sample	Per 10 percentage points	0.00 (-0.09, 0.09)	.994
Glucose	Diabetes status	Diabetes vs no diabetes	-0.42 (-0.97, 0.13)	.138
Glucose	Intervention duration	Per 10 weeks	0.10 (-0.05, 0.24)	.192
Glucose	Women in sample	Per 10 percentage points	0.05 (-0.08, 0.18)	.485
HOMA-IR	Diabetes status	Diabetes vs no diabetes	0.17 (-0.55, 0.89)	.644
HOMA-IR	Intervention duration	Per 10 weeks	-0.05 (-0.25, 0.15)	.644
HOMA-IR	Women in sample	Per 10 percentage points	-0.03 (-0.25, 0.19)	.766
LDL cholesterol	Diabetes status	Diabetes vs no diabetes	-0.48 (-1.23, 0.26)	.206
LDL cholesterol	Intervention duration	Per 10 weeks	0.13 (-0.07, 0.34)	.206
LDL cholesterol	Women in sample	Per 10 percentage points	-0.05 (-0.14, 0.04)	.290
HDL cholesterol	Diabetes status	Diabetes vs no diabetes	-0.60 (-1.32, 0.12)	.101
HDL cholesterol	Intervention duration	Per 10 weeks	0.17 (-0.03, 0.37)	.101
HDL cholesterol	Women in sample	Per 10 percentage points	0.04 (-0.05, 0.12)	.393
Triglycerides	Diabetes status	Diabetes vs no diabetes	-0.14 (-0.86, 0.57)	.693
Triglycerides	Intervention duration	Per 10 weeks	0.04 (-0.16, 0.24)	.693
Triglycerides	Women in sample	Per 10 percentage points	-0.02 (-0.10, 0.06)	.646

Note. β = standardized mean difference moderation coefficient from univariable moderation analyses. Bolded β/CI and p values indicate $p < .05$. Rows marked as not estimable were retained for transparency because the model was skipped or not sufficiently robust.

Table S7. League table with pooled standardized mean difference for different interventions and anthropometrics characteristics.

**Weight**			
**Exercise**	0.29 (0.09; 0.50)	0.53 (0.34; 0.72)	-0.47 (-0.68; -0.27)
0.26 (0.06; 0.46)	**GLP-1 RAs**	0.31 (0.11; 0.51)	-0.77 (-0.98; -0.57)
0.53 (0.34; 0.72)	0.27 (0.07; 0.47)	**GLP-1 RAs + exercise**	-1.07 (-1.28; -0.86)
-0.51 (-0.71; -0.31)	-0.77 (-0.97; -0.57)	-1.04 (-1.24; -0.83)	**Placebo**
**Waist-to-hip-ratio**			
**Exercise**	-0.16 (-0.53; 0.21)	0.20 (-0.16; 0.56)	-0.35 (-0.72; 0.02)
-0.16 (-0.53; 0.21)	**GLP-1 RAs**	0.35 (-0.01; 0.72)	-0.19 (-0.56; 0.17)
0.20 (-0.16; 0.56)	0.35 (-0.01; 0.72)	**GLP-1 RAs + exercise**	-1.01 (-1.47; -0.55)
-0.35 (-0.72; 0.02)	-0.19 (-0.56; 0.18)	-0.55 (-0.91; -0.18)	**Placebo**
**Fat-mass**			
**Exercise**	-0.05 (-0.29; 0.19)	0.38 (0.16; 0.60)	-0.60 (-0.87; -0.33)
-0.09 (-0.32; 0.14)	**GLP-1 RAs**	0.52 (0.28; 0.75)	-0.53 (-0.80; -0.26)
0.38 (0.16; 0.60)	0.47 (0.24; 0.70)	**GLP-1 RAs + exercise**	-1.05 (-1.32; -0.77)
-0.63 (-0.89; -0.37)	-0.54 (-0.80; -0.28)	-1.01 (-1.27; -0.75)	**Placebo**

Table S8. League table with pooled standardized mean difference for different interventions and glycemic profile.

**Fasting glucose**			
**Exercise**	0.33 (-0.50; 1.16)	0.04 (-0.66; 0.74)	-0.49 (-1.68; 0.70)
0.04 (-0.71; 0.80)	GLP-1 RAs	0.20 (-0.50; 0.90)	-0.33 (-1.50; 0.85)
0.18 (-0.50; 0.87)	0.14 (-0.54; 0.82)	GLP-1 RAs + exercise	-0.33 (-1.51; 0.86)
-0.30 (-1.36; 0.75)	-0.35 (-1.39; 0.70)	-0.49 (-1.52; 0.55)	Placebo
**HOMA-IR**			
**Exercise**	-0.09 (-0.41; 0.22)	0.22 (-0.10; 0.54)	-0.38 (-0.69; -0.07)
-0.08 (-0.39; 0.23)	GLP-1 RAs	0.29 (0.00; 0.57)	-0.29 (-0.59; 0.01)
0.20 (-0.11; 0.52)	0.28 (0.00; 0.57)	GLP-1 RAs + exercise	-0.60 (-0.91; -0.29)
-0.38 (-0.69; -0.07)	-0.30 (-0.60; -0.01)	-0.59 (-0.89; -0.29)	Placebo

Table S9. League table with pooled standardized mean difference for different interventions and lipid profile.

**Triglycerides**			
**Exercise**	0.19 (-0.11; 0.49)	0.03 (-0.27; 0.33)	0.16 (-0.14; 0.45)
0.17 (-0.13; 0.46)	GLP-1 RAs	-0.12 (-0.39; 0.15)	-0.03 (-0.31; 0.26)
0.04 (-0.25; 0.34)	-0.12 (-0.39; 0.14)	GLP-1 RAs + exercise	0.11 (-0.18; 0.40)
0.15 (-0.14; 0.45)	-0.01 (-0.29; 0.27)	0.11 (-0.17; 0.39)	Placebo
**LDL-C**			
**Exercise**	0.21 (-0.16; 0.58)	0.02 (-0.34; 0.38)	-0.15 (-1.10; 0.81)
0.14 (-0.21; 0.50)	GLP-1 RAs	-0.07 (-0.39; 0.25)	-0.15 (-1.16; 0.87)
0.07 (-0.28; 0.42)	-0.08 (-0.40; 0.24)	GLP-1 RAs + exercise	0.29 (-0.59; 1.18)
0.12 (-0.70; 0.93)	-0.03 (-0.84; 0.78)	0.05 (-0.75; 0.85)	Placebo
**HDL-C**			
**Exercise**	0.21 (-0.09; 0.50)	-0.10 (-0.40; 0.19)	0.27 (-0.03; 0.56)
0.17 (-0.12; 0.46)	GLP-1 RAs	-0.22 (-0.49; 0.05)	0.06 (-0.23; 0.34)
-0.05 (-0.35; 0.24)	-0.22 (-0.49; 0.05)	GLP-1 RAs + exercise	0.38 (0.09; 0.67)
0.27 (-0.03; 0.56)	0.10 (-0.18; 0.38)	0.32 (0.04; 0.61)	Placebo

Extended Methods

Data extraction

The following information was extracted based on the PICOS strategy:

Population and study characteristics

- Author, publication year, country, study design (parallel or multi-arm randomized controlled trial), sample size (total and for each intervention arm at baseline and follow-up), percentage of women, age at baseline (mean ± SD), and baseline BMI (mean ± SD).
- Studies were included if they enrolled adults (aged 18–65 years) with overweight or obesity (BMI ≥25 kg/m²) with or without type 2 diabetes. Studies with populations including participants with known psychiatric, cognitive, or non-communicable diseases were excluded.

Intervention

- GLP-1 RA type, dose and duration.
- Exercise type (aerobic, resistance, or combined exercise)
- Duration of the intervention (weeks).
- Frequency (sessions per week).
- Session duration (minutes per session).
- Exercise intensity (moderate or high), as reported by the authors.

Comparison

- Description of the comparator group: placebo, GLP-1 RAs alone, exercise alone.

Outcomes

- The primary outcomes were body weight and body composition (fat mass and waist circumference).
- Secondary outcomes included cardiometabolic parameters: fasting glucose, insulin resistance (HOMA-IR), systolic and diastolic blood pressure, triglycerides, HDL-C, and LDL-C.

For the network meta-analysis, where available, mean change-from-baseline values and corresponding standard deviations were extracted. If only pre- and post-intervention means were reported, difference scores were calculated. If only confidence intervals were available, standard deviations were estimated accordingly. If a study reported multiple time points, data from the longest follow-up closest to the end of the intervention period was extracted.

Data processing

- Continuous outcomes were standardized using the pooled pre-intervention standard deviation and converted to Hedges' g for meta-analytic purposes.
- When both change-from-baseline and post-intervention values were available, change-from-baseline values were prioritized. If only post-intervention values were reported, these were used.
- If confidence intervals were reported without standard deviations, SDs were estimated using the following formula:

$$SD = \frac{Upper\ limit - Lower\ limit}{2 \times Z} \times \sqrt{n}$$

where *Upper limit* and *Lower limit* represent the reported confidence interval bounds, Z is the z-score corresponding to the confidence level (1.96 for 95% CI), and n is the sample size.

- For each outcome, if multiple instruments or definitions were reported, prioritization of instruments was based on validation and frequency of use in the literature.
- For body composition outcomes, values from validated body composition measurement tools (e.g., DEXA, BIA) were extracted where available.
